# Sustainable development pathways for energies in Yangtze River Delta urban agglomeration

**DOI:** 10.1038/s41598-023-44727-x

**Published:** 2023-10-24

**Authors:** Chao Xu, Da Xie, Chenghong Gu, Pengfei Zhao, Xitian Wang, Yanjia Wang

**Affiliations:** 1 Department of Electrical Engineering, Shanghai Jiao Tong University, Shanghai, 200240 China; 2 Department of Electronic and Electrical Engineering, University of Bath, Bath, BA27AY UK; 3 Institute of Automation, Chinese Academy of Sciences, Beijing, 100190 China

**Keywords:** Environmental social sciences, Energy science and technology

## Abstract

The sustainable development of urban agglomerations plays a pivotal role in national and global efforts to reduce emissions. By focusing on the efficient exchange and optimization of energy consumption across various sectors, the sustainable development of energy systems within urban agglomerations can be achieved. However, the overall impact of the cross-sector energy optimization and complementarity has not been quantitatively analyzed. Here, we focused on the Yangtze River Delta (YRD) urban agglomeration in China and proposed an optimization framework for energy, environment, and economy. The framework considered four sectors: transportation sector, power sector, industry sector, and building sector, in order to determine the most sustainable development pathway for the urban agglomeration. The optimization model considers total costs and greenhouse gas emissions reduction as the objectives and utilizes technologies as constraints to optimize the pathway. We found that this optimization strategy resulted in a 53.1 billion tons increase in CO_2_ emissions reduction in the region. The results of emission reduction varied across sectors, ranging from 4.5 to 22.2 billion tons CO_2_ equivalent, and across cities, ranging from 7.1 to 4688.1 Mt. The results suggest that the core cities in the urban agglomeration can take on a leadership role. By promoting cross-sector collaboration and implementing energy recycling, the energy efficiency of surrounding cities can be greatly improved, leading to the sustainable development of the urban agglomeration.

## Introduction

Urban agglomeration^1^ is an advanced and tightly integrated urban development form^2^ which optimizes resource allocation through functional division and economic coordination. Urban agglomerations and local regions are widely selected as the primary approach to urban planning and design^3,4^ to achieve efficient, environmentally friendly, and sustainable development^5^. Cities are the fundamental entity for advancing sustainable development, and account for 60–80% of the total global energy consumption^6^ and 70–75% of carbon emissions. There are 19 urban agglomerations in China, and these agglomerations account for 73.63% of the country's total population and 32.67% of land resources. These urban areas also contribute significantly to the country's economy, generating 90.87% of its gross domestic product^7^ (GDP).

The YRD urban agglomeration is recognized as the fifth largest urban agglomeration globally^8^. It encompasses a significant portion (16.7%) of China's population and contributes to 14.2% of China's GDP^9^, despite occupying only 2.3% of its land area. The total electricity consumption was 1520.7 TWh, while the electricity generation within the region amounted to 1241.9 TWh. Notably, 18.3% of the electricity is imported from outside the YRD urban agglomeration. Additionally, the YRD urban agglomeration consumes approximately 17% of the country's total energy^10^ and emits 14% of total carbon dioxide^11^. It indicates that the YRD urban agglomeration has great potential for reducing greenhouse gas emissions. Shanghai is the core city among the 41 cities in the YRD urban agglomeration which constitutes 15.4% GDP to YRD urban agglomeration. As the core city, Shanghai facilitates the regional integration of the YRD and has the potential to enhance the efficiency and competitiveness of the surrounding cities. Besides, the interplay among cities within the urban agglomeration also fosters the integration of the urban agglomeration. The pivotal role of the core city will be crucial in driving the sustainable development of the urban agglomeration.

The Chinese government has stated that the urban agglomeration will be the predominant development model on the national strategic level^13,14^. This decision aims to facilitate the coordinated development of cities and towns. Hence, the low-carbon development of Chinese urban agglomeration will assume a pivotal role in attaining the double carbon goals proposed by the Chinese government. China has achieved urbanization at an unprecedented pace and magnitude from 36.2% in 2000 to 63.9% in 2020^15^, surpassing all other countries^16^. The Chinese government should consider the interconnected nature of cities and integrate them into urban agglomerations. China should proactively explore strategies to mitigate emissions within these urban agglomeration^17^ and endeavor to attain ambitious carbon goals. These strategies should build upon the existing emission reduction initiatives undertaken by urban agglomerations.

The synchronized operation of energy demand across various industries and sectors within urban agglomerations can lead to enhanced energy efficiency throughout the entire region, thereby facilitating the adoption of a more sustainable development model. Compared to individual sectors^18^, cross-sector energy reuse measures have the potential to achieve a further reduction of 15–36% in CO_2_ emissions. The interconnection of energy demands across different industries or sectors within cities^19–21^ or urban agglomerations^14,22–24^, including the industrial sector^25–27^, power sector^28,29^, transportation sector^30–33^, and building sector^34–37^, is crucial for achieving overall coordination. These connections can subsequently be consolidated into a more comprehensive regional or national application framework. Aggregating cities into urban agglomerations and subsequently expanding these strategies to the whole nation, facilitates a more comprehensive formulation of strategies aimed at reducing greenhouse gas emissions while circumventing a direct global^38,39^ and national^40–43^ scope. If emission reduction strategies are formulated and implemented with a focus on regional coordinated development, it becomes feasible to investigate the potential for emission reduction across various cities and their energy-consuming sectors. Urban agglomerations possess significant capabilities; however, they have been undervalued in relevant research. Therefore, this article proposes an innovative framework for optimizing the energy-environment-economy in urban clusters. While studying cross-sectoral collaborative emission reduction, this research also analyzes the pivotal role of core cities in the sustainable development of urban clusters.

**Figure 1:**
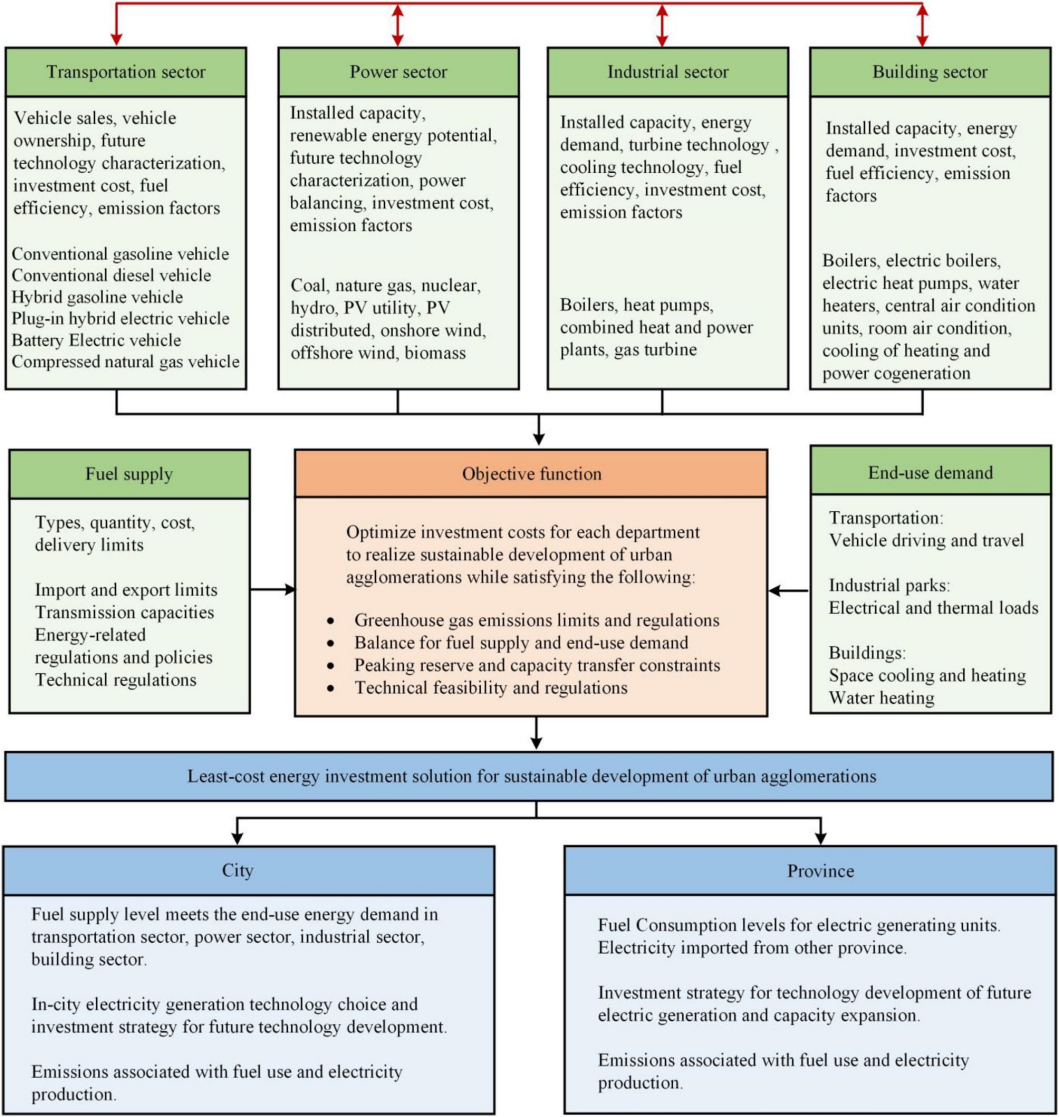
The structure of energy-environment-economy optimization for sustainable urban agglomeration development. The orange box represents the objective function, while the green boxes represent the control parameters and control objects in the transportation sector, power sector, industrial sector, and building sector. The green boxes represent the energy demand and fuel supply for end users, which are the main input variables. The blue boxes represent the output variables of the model, which are the minimum cost energy investment solutions for sustainable development in individual cities and provincial cities within the urban agglomeration. The black arrows show information flow in the model. The red arrows show energy flow in the model, which represents the flow of energy between different sectors. The electricity generated by the power sector, industry sector, and building sector is connected through the power grid. These three sectors provide the source of electricity for the transportation sector, with the power sector being the primary source of electricity.

The primary objective of this study is to develop a low-carbon development pathway and evaluate the feasibility of carbon emission reduction across different sectors within urban agglomerations. Firstly, we present a conceptual framework aimed at enhancing the optimization of the energy-environment-economy in urban agglomerations. This framework is founded upon the limitations imposed by fuel supply, end-use energy demand, and technological policy. The framework has been developed based on four distinct perspectives, namely the transportation sector, the power sector, the industry sector, and the building sector. The optimized structure for achieving sustainable development in urban agglomerations is depicted in Fig. 1. Secondly, we assess the generated scenario and technological advancements within this framework, aiming to optimize the sustainable development pathway of urban agglomerations through collaborative efforts across multiple sectors. We develop effective strategies to attain optimal objectives in terms of investment costs and emission reductions. This enables us to formulate a comprehensive strategy for energy planning and establish a sustainable development framework for energy utilization in urban agglomerations. This study aims to promote the sustainable development of urban agglomerations by addressing the question of how to achieve effective coordination among constituent cities and foster enhanced collaboration among multiple sectors. Our findings will serve as a valuable resource for the sustainable development of urban agglomerations on a global scale.

## Results

### Carbon emission reduction in the YRD urban agglomeration in 2035

**Figure 2:**
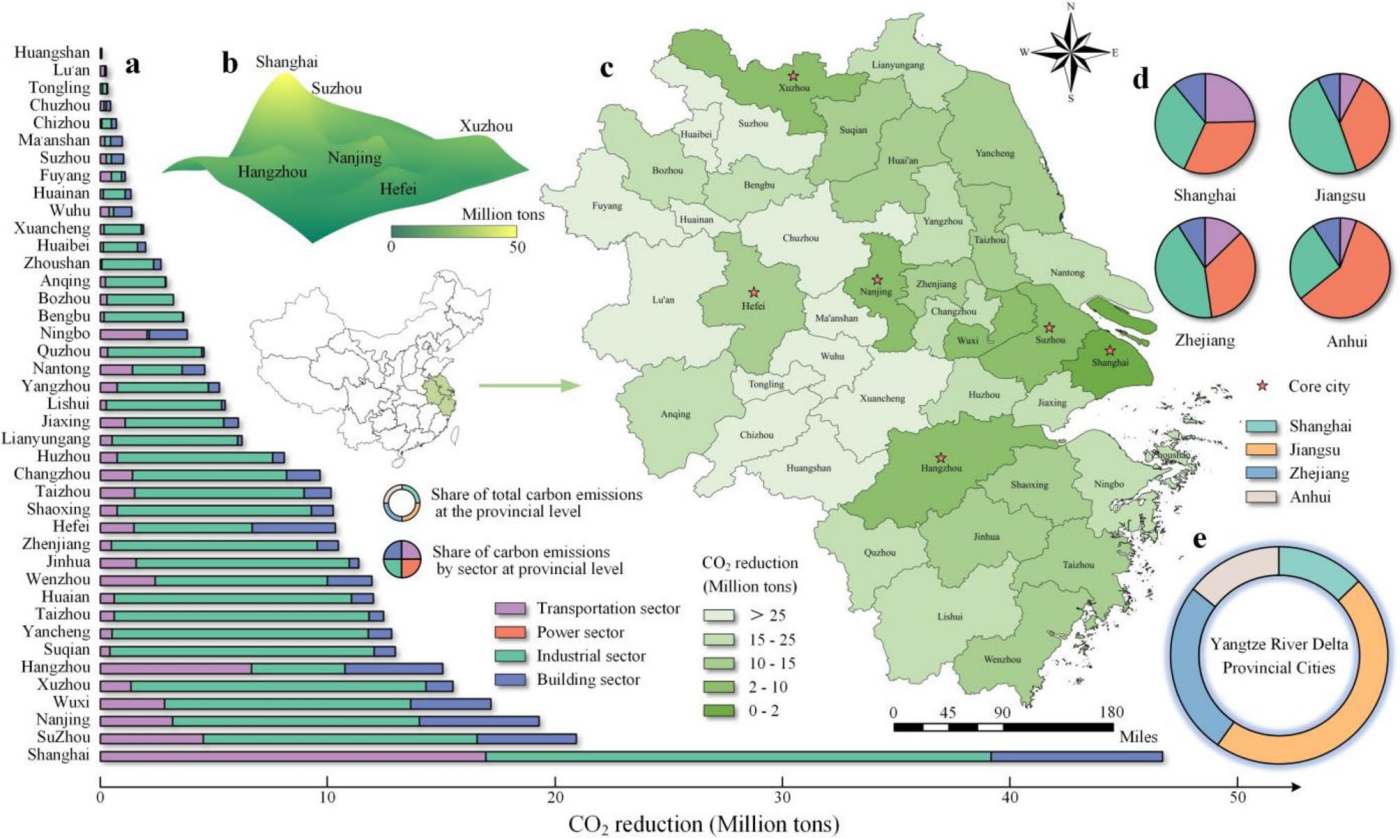
Carbon emissions reduction in the transportation sector, power sector, industrial sector, and building sectors of the Yangtze River Delta urban agglomeration. (**a**) CO_2_ emissions reduction in the transportation sector, industrial sector, and building sector of various cities within the Yangtze River Delta urban agglomeration. (**b**) A three-dimensional surface plot of total CO_2_ emission reductions from four sectors in different cities in the YRD urban agglomeration, and the peak of the surfaces are the core cities. (**c**) Total CO_2_ emissions reduction of four sectors in different cities within the Yangtze River Delta urban agglomeration is represented by the depth of color in each city, which is directly proportional to the total CO_2_ emissions of that city. (**d**) Proportion of emissions reduction by four sectors at the provincial level. (**e**) Proportion of emission reduction in the Yangtze River Delta urban agglomeration of three provinces and one city. The CO_2_ emission reduction targets shown in the figure are for the transportation sector and power sector in 2035. The industrial sector and building sector determined the annual reduction amounts of CO_2_ emissions after completing the construction of the DES. We only consider the total emission reduction amount at the provincial level for the power sector, while for the other three sectors, we consider the total emission reduction amount at the city level.

In the year 2035, the YRD urban agglomeration is projected to achieve a significant reduction in total CO_2_ emissions (53.1 billion tons). The emission reduction across various sectors varies from 4.5 to 22.2 billion tons, while the emission reduction across different cities ranges from 7.1 to 4688.1 million tons (Fig. 2a,b). The carbon emissions reduction across various sectors and cities within the YRD urban agglomeration is illustrated in Fig. 2. The industrial sector and the power sector exhibit the highest proportion of CO_2_ emission reduction, comprising 42% and 39% respectively, due to their substantial baseline emissions. Improving energy efficiency within industrial parks and enhancing the integration of renewable energy sources within the power sector have the potential to substantially mitigate CO_2_ emissions. Within an urban agglomeration, the extent to which emissions are reduced in the three sectors across various cities can also serve as an indicator of the city's economic development level. Cities that lack sufficient economic development exhibit higher levels of emissions reduction in the power sector and have a greater potential for reducing emissions in the transportation sector. Conversely, the proportion of emissions reduction in the building sector remains relatively consistent at the provincial level (Fig. 2a).

Shanghai, being the core city of the YRD, contributes to a significant reduction of 14.28% in CO_2_ emissions in the YRD urban agglomeration. At the provincial level, Suzhou and Nanjing, as the core cities of Jiangsu Province, contribute to 13.50% and 12.45% of the province's overall reduction in CO_2_ emissions, respectively. The respective proportions for Hangzhou in Zhejiang Province and Hefei in Anhui Province are 16.83% and 33.20%. This suggests that the core cities within the urban agglomeration have a significant impact on reducing CO_2_ emissions (Fig. 2d).

### Transportation sector

**Figure 3:**
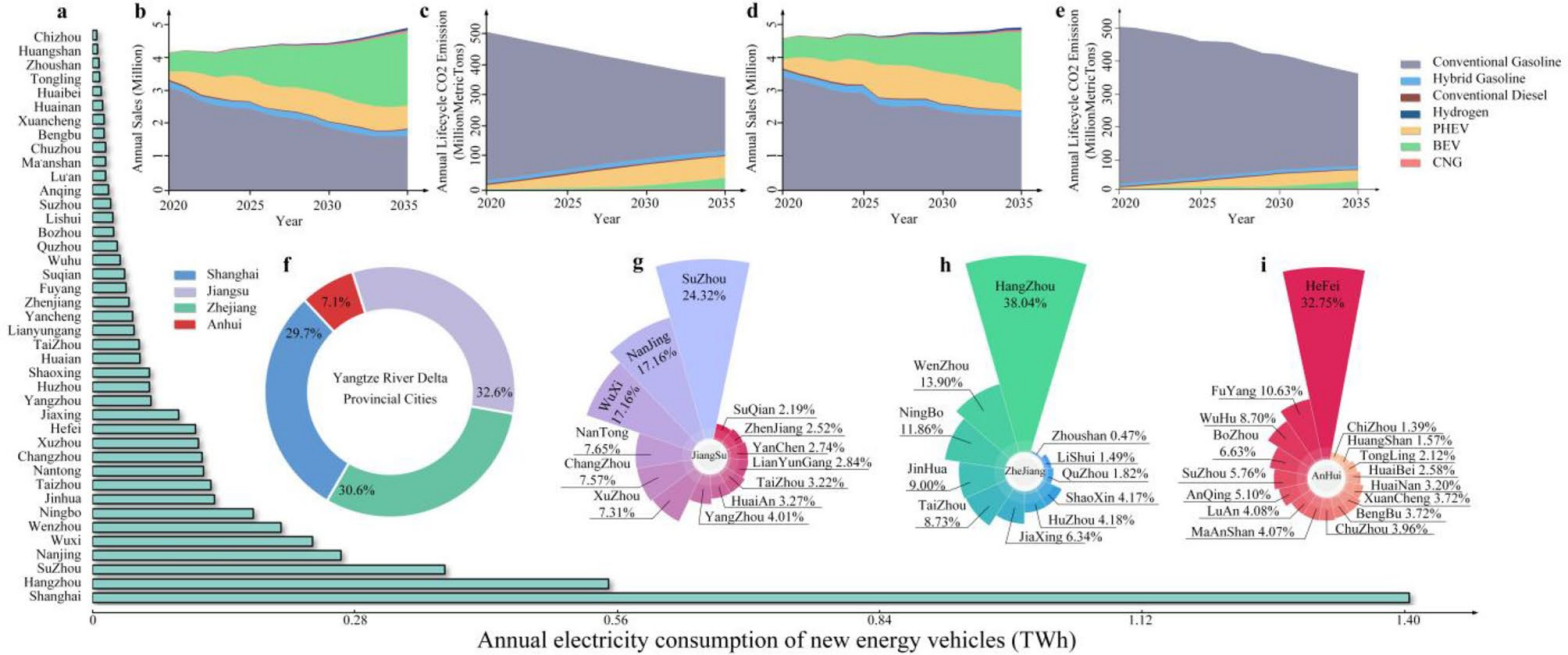
Vehicle sales and CO_2_ emissions throughout the entire lifecycle of vehicles in the Yangtze River Delta urban agglomeration. (**a**) Electricity consumption of EV in different cities within the Yangtze River Delta urban agglomeration. (**b**) Sales of different types of vehicles in high technology scenario. (**c**) CO_2_ emissions throughout the entire lifecycle of different types of vehicles in high technology scenario. (**d**) Sales of different types of vehicles in high technology scenario. (**e**) CO_2_ emissions throughout the entire lifecycle of different types of vehicles in low technology scenario. (**f**) Percentage of EV sales in the three provinces and one city of the Yangtze River Delta urban agglomeration. (**g**) Proportion of EV sales in 13 cities in Jiangsu Province. (**h**) Proportion of EV sales in 11 cities in Zhejiang Province. (**i**) of EV sales in 16 cities in Anhui Province.

The electric vehicle (EV) ownership in the YRD urban agglomeration is experiencing rapid growth, leading to a significant reduction in carbon emissions. In the policy-free scenario, our analysis reveals that the EV sales in the YRD urban agglomeration would experience a substantial increase starting from 2029 in the low technology scenario. By 2035, the light duty vehicle (LDV) fleet will consist of 1.85 million battery electric vehicles (BEVs) and 0.57 million plug-in hybrid electric vehicles (PHEVs). The average carbon emission intensity of the LDV fleet is estimated to be 195.42 g/km, and the total market share of EVs is expected to reach 48.05%, with BEVs accounting for 76.43% and PHEVs accounting for 23.57%. In the high technology scenario, it is projected that by the year 2035, EVs will hold a significant market share of 59.15%, with BEVs accounting for 76.14% and PHEVs making up the remaining 23.86%. The vehicle sales and the reduction of carbon emissions in the policy-free scenario are presented in Fig. 3. The situation in the policy scenario can be seen in Supplementary Fig. S1. Furthermore, the average carbon emission intensity associated with these EVs is estimated to be 189.48 g/km. In the high technology scenario, various factors contribute to the increased adoption of EVs. These factors include the declining prices of batteries and motors, advancements in battery energy density and generator efficiency. As a result, there has been a notable 23.11% increase in EV sales compared to the low technology scenario. This finding suggests that technological advancements can play a crucial role in boosting the EV sales without the need for additional government funding, such as subsidies for EV sales or investments in charging infrastructure. Shanghai, as the core city of the YRD urban agglomeration, holds a significant share of 29.7% in the EV sales. At the provincial level, the core cities within the Jiangsu, Zhejiang, and Anhui provinces contribute to 24.32%, 38.04%, and 32.75% of EV sales, respectively.

Policies such as infrastructure and subsidies have proven to be more effective in stimulating sales in low technology scenarios. The growth in EV sales in policy scenarios is shown in Table 1. By 2035, in the high infrastructure and low technology scenario, the number of PHEV decreased by approximately 190,000, while the number of BEV increased by 330,000. However, in the high infrastructure and high technology scenario, the increase was only 10,000 PHEVs and 140,000 BEVs. In the high subsidy scenario, the growth in EV sales is nearly two times higher in the low technology scenario compared to the high technology scenario. This disparity can be attributed to the constrained budget for subsidies, which hinders the sustainable promotion of sales in the high technology scenario. The mixed scenario of high infrastructure and high subsidies proves to be more effective in stimulating EV sales compared to a single policy scenario. In low technology scenarios, EV sales increase by 28.5% and 50% respectively, while the same trend is observed in the high technology scenarios.

**Table 1:** The growth in EV sales and their corresponding reductions in CO_2_ emissions in policy scenarios.

Technology scenario	Police scenario	Total sales over 15 years in policy scenarios (million vehicles)			Increased quantity compared to the no policy scenarios (million vehicles)			Accumulated CO_2_ reduction (Mt)
		BEV	PHEV	EV	BEV	PHEV	EV	
Low technology	High infrastructure	16.84	12.23	29.07	0.33	− 0.19	0.14	66.19
	High subsidy	16.64	12.41	29.05	0.13	− 0.01	0.12	64.19
	High infrastructure and high subsidy	16.70	12.41	29.11	0.20	− 0.02	0.18	68.63
High technology	High infrastructure	21.20	11.00	32.20	0.14	0.01	0.15	61.45
	High subsidy	21.12	11.00	32.12	0.06	0.01	0.07	53.60
	High infrastructure and high subsidy	21.21	11.06	32.27	0.16	0.07	0.23	73.43

The rise in EV sales is a direct manifestation of the penetration process of EV in the sustainable development process of urban agglomerations. The objective of sustainable development in the transportation sector is to utilize electricity as a substitute for petroleum to reduce greenhouse gas emissions. We have considered the carbon emissions associated with the entire life cycle of the vehicle fleet in the YRD urban agglomeration from 2020 to 2035. In the low technology scenario, there was a 28.8% reduction in carbon emissions in 2035 compared to 2020. Conversely, in the high technology scenario, there was an additional 5.5% reduction in carbon emissions over a 15-year period compared to the low technology scenario. In the high subsidy scenario, emissions were reduced by 54.59 and 22.93 Mt CO_2_, respectively. In the high infrastructure scenario, it was observed that there was a more significant reduction in emissions. This reduction not only contributed to the increased sales of BEVs, but also resulted in an increase in the average driving distance of both BEVs and PHEVs. As a result, there was a reduction of 66.19 Mt CO_2_ emissions for BEVs and 42.36 Mt CO_2_ emissions for PHEVs. The mixed scenario has proven to be more effective in reducing emissions when compared to a single policy scenario.

### Power sector

**Figure 4:**
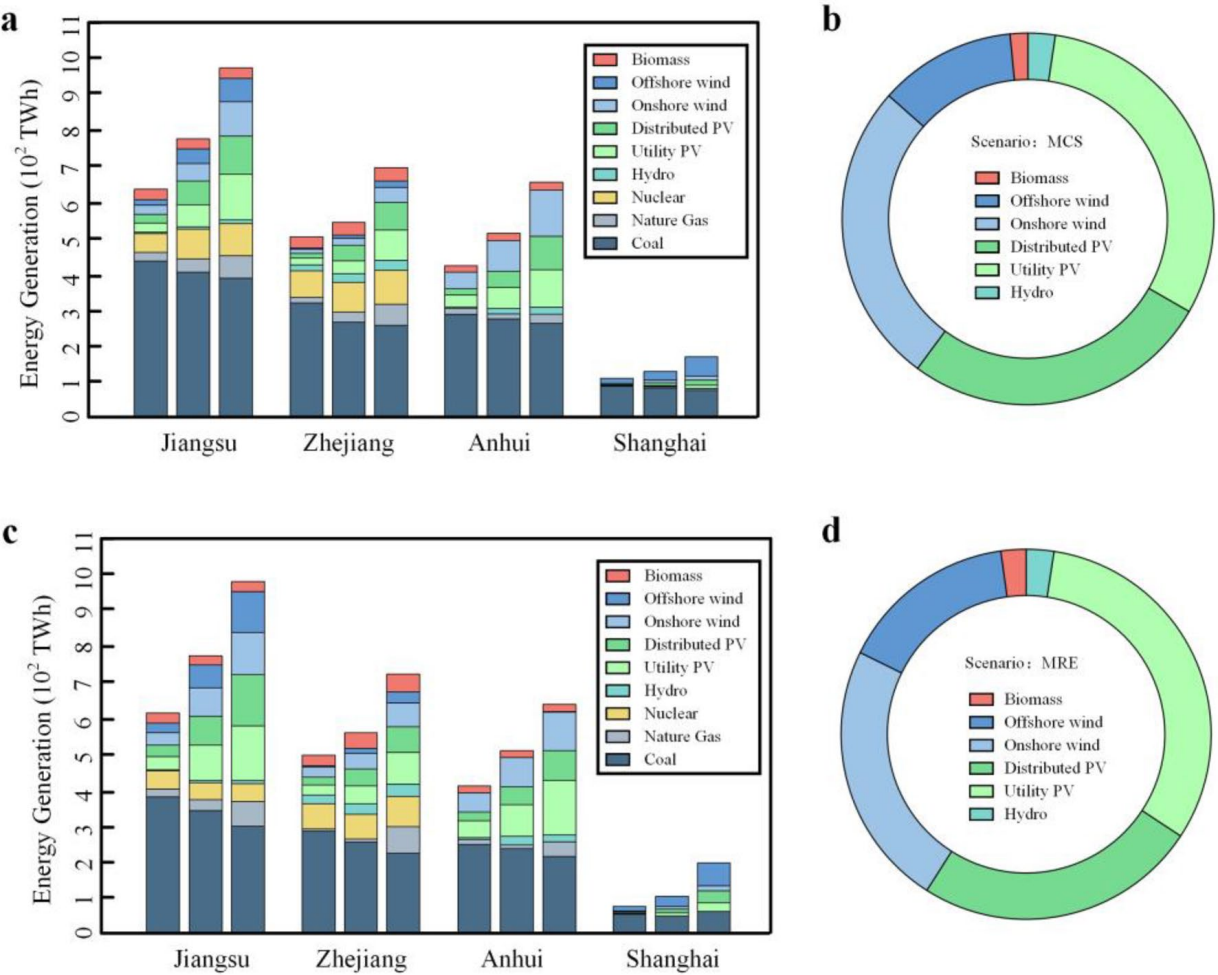
The power generation mix of the Yangtze River Delta urban agglomeration in two scenarios. (**a**) Power generation mix in MCS scenario. We considered nine types of power generation technologies, including biomass energy, offshore wind power, onshore wind power, distributed photovoltaics, utility photovoltaics, hydropower, nuclear power, natural gas power generation, and coal-fired power generation. (**b**) Increase in renewable energy generation in MCS scenario. We considered six types of power generation technologies, including biomass energy, offshore wind power, onshore wind power, distributed photovoltaics, utility photovoltaics, hydropower. (**c**) Power generation mix in MRE scenario. (**d**) Increase in renewable energy generation in MRE scenario.

In the maximize renewable energy (MRE) scenario, the generation mix of the YRD urban agglomeration will transit from fossil fuel-based energy to a power system predominantly fueled by renewable energy sources. This shift will result in a significant decrease in the proportion of thermal power generation, reducing from 82.69 to 38.78%. By 2035, utility photovoltaic is expected to dominate the renewable energy mix, with a projected share of 13.04%. This will be closely followed by onshore wind power, accounting for 11.15%. The power generation mix of the YRD urban agglomeration in two scenarios is illustrated in Fig. 4. The development model employed in the MRE scenario exhibits a positive impact on the penetration of renewable energy sources within the power supply mix. The growth rate of renewable energy generation exhibits a wide range, varying from 151 to 508%, in which Shanghai has the highest growth rate (508%). For core cities in urban agglomerations like Shanghai, it is more appropriate to focus on the development of distributed photovoltaic and offshore wind power. In the Minimum System Cost (MSC) scenario, there is a notable decrease of 21.89% in the electricity generation from wind and photovoltaic sources. This trend is observed across all individual provinces as well.

Despite the variations in scenarios, the proportion of growth in renewable energy generation remains consistent. The rise in renewable energy generation in the YRD urban agglomeration is primarily attributed to photovoltaic and wind power sources. In both the MCS and MRE scenarios, the combined share of these two sources accounts for 96.1% and 95.4% respectively. The expansion of hydropower is constrained by the availability of hydro resources in the YRD region, primarily sourced from Pumped-storage power plants located in Zhejiang Province. These power plants contribute to 68.7% of the total installed capacity of pumped storage power plants in the YRD urban agglomeration. The incremental share of offshore wind power generation in the MCS scenario and MRE scenario is 11.9% and 15.5% respectively. The analysis of the scenarios demonstrates that coastal urban agglomerations have the potential to significantly enhance the production of renewable energy through the installed capacity of offshore wind power.

By 2035, the percentage of purchased electricity gradually decreases from 22.7–53.1% to − 0.1–36.3% with the increase in renewable energy generation in the YRD urban agglomeration. Shanghai is capable of meeting its load requirements, and reducing its dependence on external power sources will lead to a more dependable power supply. However, the increasing demand for electricity may still result in power shortages as the economy continues to expand. The regions where renewable energy sources are concentrated may not always coincide with the load centers. Therefore, it is still necessary to purchase electricity from neighboring power grids to fulfill the power demand in the YRD.

In comparison to the year 2025, carbon emissions from the power sector decreased by 12.0% and 9.9% in the MRE scenario and MSC scenario respectively by 2035. The findings indicate that maximizing renewable energy utilization in the MRE scenario has successfully attained more ambitious targets for carbon emission reduction. In the power generation portfolio, despite the gradual decrease in the proportion of coal-fired power generation, it continues to be the primary contributor to carbon emissions in the YRD (2035, MRE: 88.3%, MCS: 91.6%). The decrease in carbon emissions resulting from coal-fired power generation primarily stems from variations in energy accessibility and limitations on carbon emissions. The reduction of carbon emissions from coal-fired power plants plays a critical role in promoting low-carbon development within the power sector in the YRD.

### Industrial sector and buildings sector

Currently, the annual CO_2_ emissions from 154 major national industrial parks in the YRD urban agglomeration are 30,200 tons, accounting for 16.6% of the total CO_2_ emissions in the entire urban agglomeration. Large industrial parks play a pivotal role in addressing environmental challenges within urban agglomerations. In terms of energy infrastructure, 80.5% of the units are coal-fired units. Natural gas-fired units come in second, accounting for 16.2% of the total capacity. Renewable energy driven units account for 1.3%, mainly biomass and solar energy. The remaining 2% of stocks consists of alternative fuels, including diesel, gas, waste heat, and solid waste.

**Figure 5:**
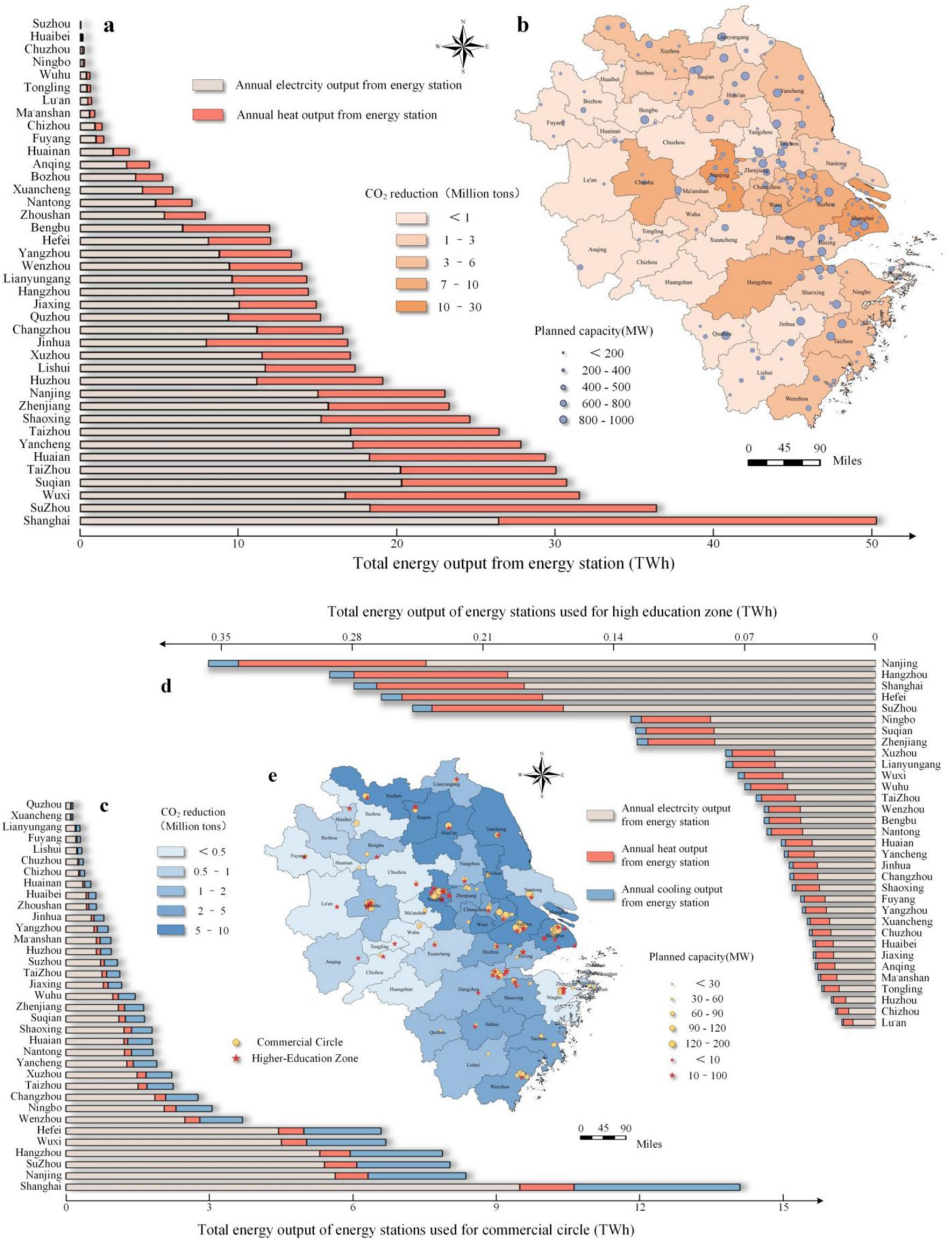

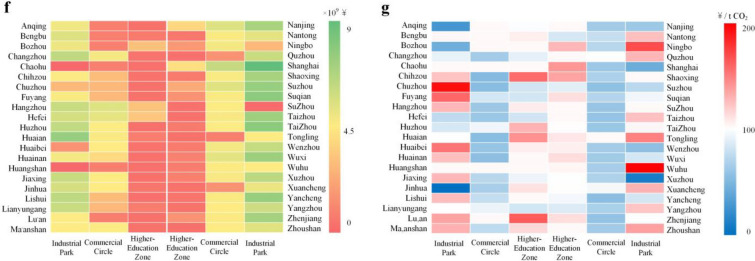
CO_2_ emissions reduction and investment costs of DES in the Yangtze River Delta urban agglomeration for industrial sector and construction sector. (**a**) The generation of electricity and heating supply from DES for industrial parks in various cities within the Yangtze River Delta city agglomeration. (**b**) CO_2_ emissions reduction in DES for industrial parks. (**c**) The generation of electricity, heating and cooling supply from DES for commercial circles in various cities within the Yangtze River Delta city agglomeration. (**d**) CO_2_ emissions reduction in DES for industrial parks. (**c**) The generation of electricity, heating and cooling supply from DES for high education zones in various cities within the Yangtze River Delta city agglomeration. (**e**) CO_2_ emissions reduction in DES for commercial circles and high education zones. The size of blue circles, yellow circles, and red pentagram symbols are proportional to the installed capacity of gas power generation devices in DES. The intensity of the background color in each city is proportional to the level of CO_2_ emissions in that city. (**f**) Investment costs for DES in industrial parks, commercial circles, and higher education zones in different cities within the Yangtze River Delta urban agglomeration. (**g**) The cost of reducing CO_2_ emissions in cities within the Yangtze River Delta urban agglomeration through the construction of energy stations.

The findings suggest that all single solutions have positive carbon reduction potentials (15.3, 7.75, 221.6 Mt CO_2_) and negative costs (49.4, 394.9, 74.36 CNY/tCO_2_). The reduction in greenhouse gas emissions achieved by the distributed energy station (DES) is illustrated in Fig. 5. S3 exhibits the highest potential for energy conservation and emission reduction because of its comprehensive renovation measures. However, it is important to note that this approach may result in a significant increase in natural gas consumption in the YRD. Furthermore, the emission reduction potential of S3 is directly correlated with the level of natural gas consumption. S1 exhibits the most notable advantages in terms of reducing CO_2_ emissions. However, the potential for emission reduction is constrained by the technical limitations of the retrofit process, thereby impeding significant scalability. S2 exhibits limited competitiveness in terms of its potential for emission reduction and cost-effectiveness. However, the reuse of municipal solid waste (MSW) also serves to mitigate environmental pressures.

Carbon emissions resulting from various sources, including electricity consumption, cooling systems, and heating processes, within the commercial circles and higher education zones in the YRD, contribute to approximately 3.73% of the overall carbon emissions in the region. The implementation of integrated energy stations can provide integrated energy services such as electricity, cooling and heating for specific business circle and higher education areas due to their concentrated energy consumption and stable demand features. Diverse cooling and heating load demands in individual business and higher education zone require corresponding construction plans and operational strategies for integrated energy stations. For centralized energy supply in commercial circle, the average installed capacity of power generation units in a single integrated energy station is 66 MW, which can reduce carbon emissions by approximately 72.22%. Furthermore, the average investment cost for carbon emission reduction is estimated to be 65.06 CNY/tCO_2_. The higher education zone has achieved a significant reduction in carbon emissions, amounting to a 71.40% decrease. This achievement has been made possible through an average investment of 90.64 CNY/tCO_2_. The investment cost associated with the construction of DES is illustrated in Fig. 5. The cumulative carbon emission reductions from the business circle and higher education zone contribute to 2.68% of the total carbon emissions in the YRD. This can be attributed primarily to the lower carbon emission factor of natural gas and the higher energy utilization rate achieved through cogeneration. The investment return period for integrated energy stations ranges from 4.5 to 6.3 years, suggesting the viability of investment and construction in this sector.

## Discussion and conclusions

We have designed a sustainable development pathway for urban agglomerations and evaluated the potential for reducing emissions in different sectors within the YRD urban agglomerations. The results showed that: (1) the industrial sector and the power generation sector have the highest proportion of CO_2_ emissions. In the future, the transportation and power generation sectors will have the greatest potential for reducing emissions. (2) The core cities within the urban clusters can play a leading role in promoting energy exchange among sectors. This can greatly enhance the efficiency of surrounding cities and contribute to the sustainable development of urban clusters.

Urban agglomeration is formed by the aggregation of multiple cities, and national efforts to mitigate emissions can also be focused on it. Urban agglomerations serve as a bottom-up intermediate hub, making the emission reduction pathway of these agglomerations crucial for both national and global emission reduction efforts. The reason why urban agglomerations can have such a positive impact is that, compared to the entire country, they can develop and implement specific emission reduction strategies based on regional coordinated development. This allows them to harness the emission reduction potential of various energy-consuming sectors both within and between cities. The interdependence of different cities, aggregated into urban agglomerations, promotes complementary advantages between cities. The core cities can collaborate with surrounding cities to improve their ability to reduce emissions.

EV has the potential to effectively address the challenges of climate change. The prioritization of promoting and implementing EV in urban areas should be a priority for the government in the transportation sector^44^. Subsidies allocated to EV and investments made in public charging stations are among the most commonly employed incentive measures^45^. In order to develop more efficient policies aimed at promoting the adoption of EV, we have employed the ADOPT model to assess the influence of subsidies, public charging infrastructure, and the demand for EV among urban residents. The evaluation criteria used in this study include the sales volume of EV and the reduction of greenhouse gas emissions. We have discovered that both subsidies and infrastructure play a significant role in effectively stimulating the sales of EV. In the high technology scenario, the most effective approach is the combination of subsidies and investment. In the low technology scenario, where battery costs are higher, investing in a single public charging station is more effective. This study aims to examine the vehicle preferences of urban residents in the YRD, taking into account various policy scenarios. We specifically focus on the technical features of EV and the availability of public charging stations. In the context of advancing diverse interconnected technologies, the implementation of car subsidies and investments in public charging stations can significantly facilitate the widespread adoption of EV within urban agglomeration. When formulating policies to promote the adoption of EV^46^, it is crucial for the government to take into account various factors. These factors include the technological background^47^, gasoline prices^48^, electricity prices, the purchasing psychology of urban residents^49^, and the availability of charging facilities^50^.

In order to attain sustainable development in the power sector within urban agglomeration, it is imperative to prioritize wind power and photovoltaic resources as the primary sources, while utilizing various flexible power generation resources as supplementary means. Shanghai, being the core city of the YRD urban agglomeration, has the lowest levels of carbon emissions and assumes a leadership role in promoting sustainable development within the entire urban agglomeration. For urban agglomerations, it is of utmost importance to prioritize the development of distributed photovoltaics in core cities, as this will effectively mitigate the wastage of land resources. Correspondingly, it is imperative for coastal cities within urban agglomerations to actively pursue the development of offshore wind power. Given the significant adoption of renewable energy sources, there has been a substantial increase in investments in energy storage systems. These investments aim to address the safety concerns associated with maintaining real-time power balance and minimum system inertia^51,52^. The production capacity of wind power, photovoltaic, and energy storage devices should be sufficient to meet the demands of energy transformation. Ensuring the integrity of the industrial chain, including its upstream and downstream components, as well as the supply chain, is crucial for achieving sustainable development in the field of energy transformation. Simultaneously, the integration of renewables has resulted in a notable escalation in the supply cost of the power system. This necessitates the establishment of a comprehensive electricity market mechanism, encompassing both spot market and auxiliary services market^53^, to ensure equitable distribution of profits.

The energy infrastructure of industrial parks that predominantly depend on coal as their primary fuel source can significantly contribute to the mitigation of greenhouse gas emissions. To mitigates the reliance on coal within industrial parks and enhance the energy utilization efficiency of their energy infrastructure^27^, we have developed comprehensive emission reduction strategies tailored specifically for these industrial parks. These plans offer practical support in advancing the sustainable development in the YRD urban agglomeration, and can be effectively implemented in other urban agglomeration within China as well as in other developing nations. This article exclusively focuses on the objective of reducing emissions through the optimization of energy infrastructure within industrial parks. However, it is important to acknowledge the existence of a symbiotic relationship between cities and industries. The utilization of low-grade waste heat produced by industrial processes has the potential to be reused for urban heating and other applications^54,55^. The promotion of energy transformation necessitates the integration of the heat network and the power grid^56^.

Traditional energy system planning is constrained the restriction on a singular form of energy, thereby preventing the complementary between diverse energy sources. In the YRD urban agglomeration, there is a significant demand for heating and cooling in commercial circles and higher education zone. Therefore, it is imperative to establish DES in close proximity, employing combined cooling, heating, and power (CCHP) technology in order to accomplish cascading utilization of energy. A highly efficient and scalable distributed CCHP system is a significant strategy for achieving building energy conservation and maximizing the efficient utilization of natural gas. The residential load in the YRD urban agglomeration currently lacks the prerequisites for centralized energy supply. However, a comprehensive investigation into centralized heat supply in residential areas, similar to the northern urban agglomerations like Beijing-Tianjin-Hebei, can be pursued for further research. Natural gas serves as a substitute for coal and contributes to both energy conservation and the reduction of emissions. One crucial approach to attaining sustainable development in urban agglomerations is through the penetration of the industrial and commercial fuel market as a substitute for boiler fuel. If coal-fired boilers are converted to natural gas, the cost of unit heat energy will increase by more than threefold^57^, which leading to financial burdens for consumers. Hence, in the case of large-scale industries and buildings, such as industrial parks, commercial circles, and higher education zones, the implementation of DES can be considered as a viable solution to promote sustainable development. These integrated energy stations can effectively enhance energy efficiency, mitigate the impact of high energy prices, and reduce overall costs.

The urban agglomeration's core cities exhibit a significant reduction in total emissions, whereas the non-core cities possess a greater potential for emission reduction. Hence, it is imperative to harness the full potential of core cities in the urban agglomeration to effectively reduce emissions^58^. This can be achieved by bolstering their leadership and driving capabilities, fostering better communication and cooperation between core cities and surrounding cities^59^, promoting the utilization of complementary strengths among cities, and collaborating with surrounding cities to enhance their capacity for emission reduction^60^. We have formulated a sustainable and environmentally-friendly development strategy for the YRD urban agglomeration. This development model has the potential to be replicated in other urban agglomerations.

## Methods

### Scenario design

We have designed four main scenarios for the transportation sector: the no-policy scenario and three policy scenarios based on a fixed total budget. In the no-policy scenario, it is assumed that there are no additional subsidies or policy support. The three policy scenarios include the deployment of public charging stations, vehicle subsidies, and a mixed of both. These four scenarios are evaluated based on two technology scenarios: low technology scenario and high technology scenario, mainly estimating the parameters of electric vehicle-related technologies, such as battery and motor.

Two distinct scenarios are established for the power sector in urban agglomerations. One development model aims to maximize renewable energy generation per unit cost, with the objective of maximizing the renewable energy generation. The other is a development model with a focus on economics, aiming to minimize system costs.

We have put forth three CO_2_ reduction schemes^61^ that are applicable to most industrial parks, aligning with the objectives of the national energy strategy. S1: Transform the extraction condensing unit or pure condensing unit of a steam turbine into a back pressure unit. S2: Replace coal-fired boilers with waste incineration boilers. S3: Replace coal-fired units with gas-fired units. S1 represents a technological advancement, whereas S2 and S3 focuses on decreasing the reliance on coal consumption. Each industrial park will undergo an individual assessment of its existing units and energy usage in order to determine the most appropriate emission reduction scheme.

### The ADOPT model

We employ The Automotive Deployment Options Projection Tool^62,63^ (ADOPT) to simulate the adoption of new energy vehicles in the context of sustainable development in the YRD urban agglomeration from 2020 to 2035. This study employs a quantitative approach to analyze the influence of technological advancements on the sales of automobiles, consumption of fossil energy, and carbon emissions.

The ADOPT model incorporates the inclusion of established car brands, models, and their respective vehicle parameters in order to comprehensively depict the present market scenario and accurately simulate existing vehicles. Based on this information, new car models that may emerge in the future are developed. Simultaneously, the Future Automotive System Technology Simulator (FASTS) is utilized to assess the enhancement in performance and the technical cost of crucial car components, including batteries and engines, within the ADOPT model.

When estimating car sales, the weighted values of various key attributes of the vehicles are determined using historical sales data. These attributes include price, fuel cost, vehicle size, and power system. Additionally, consumer psychology is taken into consideration during the estimation process. Sales are subject to fluctuations based on income levels, and the accuracy and dependability of projected sales data for future years are verified by comparing it with actual historical sales data. When assessing the mileage of cars throughout their lifecycle, various factors are taken into account, including the impact of aging and scrapping on decreased mileage, as well as individual driving mileage requirements. In conjunction with the annual sales volume of new cars, calculations are made to determine fuel consumption and CO_2_ emissions. The supplementary materials S2 provide an introduction to the technical parameter settings associated with this model.

### Power development planning model for urban agglomeration

Two optimization models have been developed to address various scenarios. One development model is oriented towards minimizing the total system cost and maximizing economic benefits. The primary aim of the objective function is to minimize the cumulative expenses incurred from investment, maintenance, and operation over the designated planning period. The objective function is as follows:

$$ \min C = \sum\limits_{t = 1}^{{n_{t} }} {\sum\limits_{i = 1}^{{n_{i} }} {C_{PS\_I}^{i,t} } + C_{PS\_O\& M}^{i,t} } $$

where, $C_{I}^{i,t}$, $C_{PS\_O\& M}^{{\text{i,t}}}$ represent the investment cost, maintenance cost and operating cost of the *i*-th city in year *t*, respectively; The planned cities include Shanghai, Jiangsu Province, Zhejiang Province, and Anhui Province, with a planning period from 2020 to 2035.

The second model focuses on optimizing the generation of renewable energy while minimizing the unit cost. The objective function of the system is as follows:

$$ \min E = \frac{{\sum\limits_{type} {\sum\limits_{i = 1}^{{n_{i} }} {\Delta C{\text{ap}}_{type}^{i,t} } } }}{{\sum\limits_{type} {\sum\limits_{i = 1}^{{n_{i} }} {C_{PS\_I}^{i,t} } } }},type \in \left\{ {\text{renewable energy generation}} \right\} $$

where, $\Delta C{\text{ap}}_{type}^{i,t}$ represents new installed capacity of renewable energy for the *i*-th city in year *t*; *type* represents the types of renewable energy.

$$ C_{PS\_I}^{i,t} = \sum\limits_{type} {\sum\limits_{i = 1}^{{n_{i} }} {\sum\limits_{t = 1}^{{n_{t} }} {\frac{{r_{DR} }}{{1 - (1 + r_{DR} )^{ - t} }}} } } \cdot p_{I}^{type} \cdot \Delta Cap_{type}^{i,t} $$

$$ C_{PS\_O\& M}^{i,t} = \sum\limits_{type} {\sum\limits_{i = 1}^{{n_{i} }} {p_{O\& M}^{type} \cdot Cap_{type}^{i,t} } } \,\,type \in \left\{ {generator,ESS} \right\} $$

where, *r*_*DR*_ is the discount rate; $p_{I}^{type}$ and $p_{O\& M}^{type}$ represent the investment, operation and maintenance costs corresponding to different types of power sources per unit capacity, respectively; $\Delta Cap_{type}^{i,t}$ represents the installed capacity of different types of power sources added to *i*-th city in year *t*; $Cap_{type}^{i,t}$ represents the installed capacity of different types of power sources for *i*-th city in year *t*.

### Geographic information system-based energy consumption database

The energy consumption database presented in this study is constructed upon three distinct entities, namely industrial parks, commercial circles, and higher education zones. The database comprises geographical location data, electricity load data, heating demand data, and cooling demand data. The energy data pertaining to industrial parks in the YRD is derived from existing literature sources. The geographical coordinates of these parks are obtained using Google Earth. The study encompasses a total of 154 national-level industrial parks^64^. The building outlines and the latitude and longitude coordinates of 1542 commercial districts in the YRD were acquired from commercial map software (BIGEMAP). However, it should be noted that the majority of these commercial circles are characterized by small scale and are not conducive to energy-saving and emission reduction through centralized energy supply. We have identified 148 business zones that are deemed suitable for centralized energy supply, taking into consideration the size of these commercial circles. Some business zones in close proximity were consolidated into a single business zone in order to facilitate centralized energy supply. Taking into account the agglomeration characteristics and quantity restrictions of higher education zones, we compiled a comprehensive list of 54 higher education zones in the YRD through direct statistical analysis.

Based on the aforementioned list of industrial parks, commercial circles, and higher education zones, we have collected energy usage data from various sources. The primary sources of data encompass statistical yearbooks, statistics derived from the Chinese power industry, reports on commercial complexes released by local governments, public disclosure of energy consumption by universities, pertinent websites, and literature. Based on the aforementioned information, we have successfully developed a comprehensive energy consumption database that encompasses 154 industrial parks, 148 business zones, and 54 higher education zones. The database has been integrated into a Geographic Information System (GIS), thereby creating a geographic information database that encompasses 356 regions. This study will provide the basis for future research on the construction and optimization of energy stations, focusing on their operation.

### Distributed energy station construction and operation optimization model

The investment and operational strategy of the respective energy station is formulated by considering the cooling and heating energy demand of a specific area, with the objective of minimizing the overall cost. The objective function includes the construction cost of the energy station and the revenue generated from its operation. The cost of construction encompasses the expenses associated with the investment, operation, and maintenance of the energy station. The revenue generated by the energy station comprises the income derived from the production of electricity, cooling, and heating.

$$ {\text{F = min C}}_{DES}- {\text{ R}}_{DES} $$

$$ {\text{C}}_{DES} = C_{DES\_I} + C_{DES\_O\& M} $$

$$ {\text{R}}_{DES} = R_{e} + R_{{\text{h}}} + R_{c} $$

where, C_DES_ is the total cost of investment in energy stations, R_DES_ is the total revenue from the operation of energy stations, C_*DES_I*_ is the investment costs of the energy station, C_*DES_O&M*_ is the operation and maintenance costs of the energy station; R_e_ is the revenue from electricity sales, R_h_ is the revenue from heat sales; R_c_ is the revenue from cooling sales.

$$ C_{DES\_I} = C_{DES\_e} + C_{DES\_i} + C_{DES\_c} + C_{DES\_o} $$

$$ C_{{{\text{DES\_e}}}} = \sum\limits_{type} {\sum\limits_{i = 1}^{{n_{i} }} {\sum\limits_{j = 1}^{{n_{j} }} {\sum\limits_{m = 1}^{{n_{m} }} {Cap_{m}^{i,j} \cdot p_{m,type}^{i,j} \cdot \delta_{m} \cdot N_{m} } } } ,type \in \left\{ {unit,control,auxiliary} \right\}} $$

$$ C_{DES\_i} + C_{DES\_c} + C_{DES\_o} = \sum\limits_{{type_{1} }} {\lambda_{{type_{1} }} \cdot \sum\limits_{i = 1}^{{n_{i} }} {\sum\limits_{j = 1}^{{n_{j} }} {\sum\limits_{m = 1}^{{n_{m} }} {C{\text{ap}}_{m}^{i,j} } } } } + \sum\limits_{{type_{2} }} {\kappa_{{type_{2} }} \cdot C_{DES\_I} } $$

$$type_{1} \in \left\{ {installation,other} \right\},type_{4} \in \left\{ {construction,other} \right\}$$

$$ \begin{gathered} C_{DES\_O\& M} = p_{NG} \cdot \eta \cdot \sum\limits_{i = 1}^{{n_{i} }} {\sum\limits_{j = 1}^{{n_{j} }} {\sum\limits_{m = 1}^{{n_{m} }} {\left( {P_{m,e}^{i,j} + P_{m,h}^{i,j} + P_{m,c}^{i,j} } \right)} } } \\ \quad+ \sum\limits_{{type_{3} }} {\lambda_{{type_{3} }} \cdot \sum\limits_{i = 1}^{{n_{i} }} {\sum\limits_{j = 1}^{{n_{j} }} {\sum\limits_{m = 1}^{{n_{m} }} {C{\text{ap}}_{m}^{i,j} } } } } + \sum\limits_{{type_{4} }} {\kappa_{{type_{4} }} \cdot C_{DES\_I} } \\ \end{gathered} $$

$$type_{3} \in \left\{ {salary,repairs,material,rent} \right\},type_{4} \in \left\{ {insurance,other} \right\}$$

where, C_*DES_e*_ is the cost of purchasing generator unit, C_*DES_i*_ is the cost of equipment installation, C_*DES_c*_ is the cost of construction, C_*DES_o*_ is other cost, C_*DES_O&M*_ is the cost of operation and maintenance; $Cap_{m}^{i,j}$ is the installed capacity of the *m*-th type of generator unit in the *j*-th commercial circle or higher education zone in the *i*-th city, $p_{m}^{i,j}$ is the unit installed capacity price of the *m*-th type of generator unit in the *j*-th commercial circle or higher education zone in the *i*-th city, $P_{m,h}^{i,j}$, $P_{m,e}^{i,j}$, $P_{m,c}^{i,j}$ represents the power generation, heating, and cooling capacity of the *m*-th generation unit in the *j*-th commercial circle or higher education zone in the *i*-th city, respectively; *δ*_*m*_ is 0 or 1; *N*_*m*_ is number of generation units of type *m; λ*_*type*_ and *κ*_*type*_ are coefficient of cost calculation; *p*_*NG*_ is the price of nature gas; *η* is the energy use efficiency of the generating unit.

$$ \lambda_{m,CHP} = {{P_{m,h}^{i,j} } \mathord{\left/ {\vphantom {{P_{m,h}^{i,j} } {P_{m,e}^{i,j} }}} \right. \kern-0pt} {P_{m,e}^{i,j} }} $$

$$ P_{m,h}^{i,j} + P_{m,e}^{i,j} = Cap_{m}^{i,j} \cdot h_{m}^{i,j} \cdot N_{m} $$

$$ P_{m,c}^{i,j} = \lambda_{hc} \cdot \eta_{hc} \cdot P_{m,h}^{i,j} $$

$$ {\text{R}}_{DES} = \sum\limits_{i = 1}^{{n_{i} }} {\sum\limits_{j = 1}^{{n_{j} }} {\sum\limits_{m = 1}^{{n_{m} }} {\left( {P_{m,e}^{i,j} \cdot p_{e} + P_{m,h}^{i,j} \cdot p_{h} + P_{m,c}^{i,j} \cdot p_{c} } \right)} } } $$

where, *λ*_*m,CHP*_ is the thermoelectric ratio of the m-th generator unit; $h_{m}^{i,j}$ is the annual number of operating hours of the *m*-th type of generating unit in the *j*-th commercial circle or higher education zone in the *i*-th city; *λ*_*hc*_ is the efficiency of waste heat refrigeration; *η*_*hc*_ is the waste heat ratio used for refrigeration; *p*_*e*_, *p*_*h*_, *p*_c_ is the price of electricity, heat, and cooling.

### Supplementary Information

Supplementary Information 1.

Supplementary Information 2.

## Data Availability

The Automotive Deployment Options Projection Tool is an open-source integrated assessment model, available at: https://www.nrel.gov/transportation/adopt-download-confirmed.html. The optimization model is conducted using MATLAB 2021a. Figures are created using Microsoft Visio professional 2019. Computer code is available from the corresponding author on reasonable request. All data generated or analyzed during this study are included in this published article and its supplementary information files.
