# Supplementary material for: Sustainable development pathways for energies in Yangtze River Delta urban agglomeration
Source: Sci Rep. 2023 Oct 24;13:18135. doi: 10.1038/s41598-023-44727-x (PMC10598035; doi:10.1038/s41598-023-44727-x)
Supplement: Supplementary file 1 — Supplementary Information 1. [file 41598_2023_44727_MOESM1_ESM.docx]

**Supplementary information for Sustainable Development Pathways for Energies in Yangtze River Delta Urban Agglomeration**

**Supplementary Note 1： Parameters and results of ADOPT model**

Technical parameters related to electric vehicles

The technical parameters of the ADOPT model are shown in the supplementary table 1. The price of batteries is based on Bloomberg NEF's predictions, which is only the expected average price. Motor prices and battery energy density are derived from data provided by the US Department of Defense and the Department of Energy.

**Supplementary Table 1. Electric vehicle related parameters**

**in Low Technology and High Technology scenarios from 2020 to 2035**

| Parameter | Scenario | 2020 | 2025 | 2030 | 2035 |
| --- | --- | --- | --- | --- | --- |
| Battery Pack Price($/kWh) | Low Technology | 200 | 150 | 125 | 100 |
|  | High Technology | 200 | 100 | 60 | 45 |
| Motor Price($/kW) | Low Technology | 34.80 | 25.00 | 20.60 | 18.50 |
|  | High Technology | 34.80 | 18.10 | 12.10 | 9.50 |
| BEV energy density(Wh/kg) | Low Technology | 175 | 230 | 310 | 400 |
|  | High Technology | 175 | 200 | 230 | 260 |
| PHEV energy density(Wh/kg) | Low Technology | 70 | 90 | 110 | 130 |
|  | High Technology | 70 | 110 | 125 | 145 |
| Power to weight ratio(W/kg) | Low Technology | 1125 | 1350 | 1500 | 1700 |
|  | High Technology | 1125 | 1440 | 1600 | 1800 |

**Supplementary Table 2. Parameters of car engine from 2020 to 2035**

| Parameter | 2020 | 2025 | 2030 | 2035 |
| --- | --- | --- | --- | --- |
| SI peak efficiency(%) | 0.36 | 0.38 | 0.40 | 0.42 |
| CI peak efficiency(%) | 0.42 | 0.43 | 0.44 | 0.45 |
| Atkinson cycle  peak efficiency(%) | 0.39 | 0.40 | 0.41 | 0.42 |
| SI engine price($/kW) | 45 | 45 | 45 | 45 |

The ADOPT model determines the number of car ownership based on the simulated sales data of cars over the years, combined with the aging and scrapping of vehicles. The number of charging stations required depends on the number of battery electric vehicles (BEV) and plug-in hybrid electric vehicles (PHEV). In low technology scenario, it corresponds to a fleet of around 21 million, in high technology scenario, it corresponds to a fleet of around 24 million, and in low technology scenario, it corresponds to 58% of BEV and 42% of PHEV, In high technology scenario, it corresponds to 69% of BEV and 31% of PHEV. In 2020, the national vehicle to pile ratio was 3.05:1. According to the requirements of the National Energy Administration's charging pile construction task, the vehicle to pile ratio in 2030 was 2:1. Based on this growth rate, we obtained the vehicle to pile ratio of electric vehicles in 2025 and 2035, and estimated the required number of charging piles. The ADOPT model will use the electric vehicle fleet size and the number of charging piles to evaluate the availability of charging stations, and then determine the impact of charging stations on the sales and ownership of electric vehicles.

**Supplementary Table 3. Number of BEV and PHEV ownership and required charging stations**

| Parameter | Scenario | 2020 | 2025 | 2030 | 2035 |
| --- | --- | --- | --- | --- | --- |
| BEV fleet | Low Technology | 573666 | 4044322 | 8590475 | 12259223 |
|  | High Technology | 548734 | 4275092 | 10253532 | 16719231 |
| PHEV fleet | Low Technology | 160275 | 3197716 | 7619199 | 8813430 |
|  | High Technology | 130182 | 3386485 | 6928162 | 7349826 |
| Required chargers | Low Technology | 240636 | 2896815 | 8104837 | 11390623 |
|  | High Technology | 222595 | 3064630 | 8590847 | 13010301 |

According to the latest policy of the Chinese government, subsidies for new energy vehicles will expire on December 31, 2022, and vehicles registered after December 31, 2022 will no longer receive subsidies. However, although there is no national subsidy, in order to further promote the consumption of new energy vehicles, local governments in many regions have introduced corresponding preferential policies. In 2023, purchasing new energy vehicles that meet the requirements can receive a subsidy of $842 to $1404. In the scenario of high subsidies, we assume that the subsidy will continue to exist. The following table lists the subsidy costs for electric vehicles in key years.

**Supplementary Table 4. Subsidy cost for purchasing BEV and PHEV**

| Parameter | 2020 | 2025 | 2030 | 2035 |
| --- | --- | --- | --- | --- |
| BEV | 3000 | 1500 | 700 | 350 |
| PHEV | 1200 | 600 | 300 | 0 |

The left figure below shows the annual sales of vehicles in the Yangtze River Delta urban agglomeration under different scenarios, while the right figure shows annual lifecycle CO_2_ emission.

**Scenario: low technology & no policy**

|  |  |
| --- | --- |

**Scenario: high technology & no policy**

|  |  |
| --- | --- |

**Scenario: low technology & high infrastructure**

|  |  |
| --- | --- |

**Scenario: low technology & high subsidy**

|  |  |
| --- | --- |

**Scenario: low technology & high infrastructure & high subsidy**

|  |  |
| --- | --- |

**Scenario: high technology & high infrastructure**

|  |  |
| --- | --- |

**Scenario: high technology & high subsidy**

|  |  |
| --- | --- |

**Scenario: high technology & high infrastructure & high subsidy**

|  |  |
| --- | --- |

**Supplementary Figure 1 Vehicle sales and CO2 emissions throughout the entire lifecycle of vehicles in the various scenarios.**

**Supplementary Note 2：Optimization model for power sector**

Optimization Models for MCS and MRE Scenarios in the Power Sector

Two optimization models have been developed to address various scenarios. One development model is oriented towards minimizing the overall system cost and maximizing economic benefits. The primary aim of the objective function is to minimize the cumulative expenses incurred from investment, maintenance, and operation over the designated planning period. The objective function is as follows:

where, $C_{I}^{i,t}$、represent the investment cost, maintenance cost and operating cost of the *i*-th city in year *t*, respectively; The planned cities include Shanghai, Jiangsu Province, Zhejiang Province, and Anhui Province, with a planning period from 2020 to 2035.

The second model focuses on optimizing the generation of renewable energy while minimizing the unit cost. The objective function of the system is as follows:

$$\min E=\frac{\sum_{type} \sum_{i=1}^{n_{i}} \Delta C\text{ap}_{type}^{i,t}}{\sum_{type} \sum_{i=1}^{n_{i}} C_{PS\_I}^{i,t}},type\in\left\{\text{renewable energy generation}\right\}$$

where, $\Delta C\text{ap}_{type}^{i,t}$ represents new installed capacity of renewable energy for the *i*-th city in year *t*; *type* represents the types of renewable energy.

$$C_{PS\_I}^{i,t}=\sum_{type} \sum_{i=1}^{n_{i}} \sum_{t=1}^{n_{t}} \frac{r_{DR}}{1-(1+r_{DR})^{-t}}\cdot p_{I}^{type}\cdot\Delta Cap_{type}^{i,t}$$

$$type\in\left\{generator,ESS\right\}$$

where, *r_DR_* is the discount rate; $p_{I}^{type}$ and represent the investment, operation and maintenance costs corresponding to different types of power sources per unit capacity, respectively; $\Delta Cap_{type}^{i,t}$ represents the different types of installed power capacity that were incorporated into *i*-th city in year *t*; $Cap_{type}^{i,t}$ represents the installed capacity of different types of power sources for *i*-th city in year *t*.

Constraints

Constraints on renewable energy capacity that can be developed

The potential for exploiting resources in various provinces and regions is subject to limitations for hydropower units, wind power units, photovoltaic units, and biomass energy units. This constraint can be formulated as follows:

$$Cap_{type}^{i}\leq\bar{C}ap_{type}^{i},type\in\left\{Hydro,PV,W\text{ind power,Biomass}\right\}$$

where, $Cap_{type}^{i}$ represents the installed capacity of renewable energy corresponding to the *i*-th city; $\bar{C}ap_{type}^{i}$ represents the upper limit for the installed capacity of renewable energy corresponding to the *i*-th city.

Power supply and demand balance constraints

$$\left(1-\eta_{loss}^{i}\right)\cdot\sum_{type} P_{type}^{i}=P_{load}^{i},type\in\left\{generator\right\}$$

where, $\eta_{loss}^{i}$ represents the line loss rate of the *i*-th city; $P_{type}^{i}$ represents the power generation of the corresponding type of power source in the *i*-th city; $P_{load}^{i}$ represents the load for the *i*-th city.

Natural gas resource constraints

The availability of natural gas resources in the Yangtze River Delta, and indeed in China as a whole, is constrained, with a heavy dependence on imports. Furthermore, the supply of natural gas resources cannot surpass the capacity of the existing natural gas power generation resources in the region.

$$\sum_{i=1}^{n_{i}} \eta_{m}\cdot P_{NG,i,t}\leq\Gamma_{y}+\Gamma_{y}^{import}+\Gamma_{y}^{\exp ort}$$

$$0\leq\Gamma_{y}^{import}\leq\bar{\Gamma}_{y}^{import}$$

$$0\leq\Gamma_{y}^{export}\leq\bar{\Gamma}_{y}^{export}$$

where, *P_NG,i,t_* represents the natural gas power generation of the *i*-th city in the *t*-th year; $\eta_{m}$ represents the natural gas consumed per unit of natural gas power generation; $\Gamma_{y}$ represents the natural gas supply for natural gas power generation; $\Gamma_{y}^{import}$ and $\Gamma_{y}^{\exp ort}$ represents natural gas imports and exports, respectively.

Water resource limitations

The water consumption for power generation in each province during the planning stage must not exceed the prescribed limit for power generation

$$\sum_{type} \sum_{i=1}^{n_{i}} \omega_{type}\cdot P_{type,i,t}\leq\bar{W}_{i}$$

where, *ω_type_* represents the water consumption per unit of electricity generated by the corresponding power generation technology; *P_type,i,t_* represents the power generation capacity of the *i*-th city in year *t*, which is the upper limit of water resources used for power generation.

Constraints on the upper limit of carbon emissions

The CO2 emissions generated by the power sector need to align with the specified carbon reduction objectives.

$$\sum_{type} \sum_{i=1}^{n_{i}} e_{type}P_{type}^{i,t}\leq E^{i,t},type\in\left\{generator\right\}$$

where, *e_type_* represents the carbon emission coefficient of the corresponding type of power supply; $P_{type}^{i,t}$ represents the power generation of the corresponding type of power source in the *i*-th city in the *t*-th year; *E^i,t^* represents the carbon reduction target for the *i*-th city in the *t*-th year.

**Supplementary Table 6. Generation Combination of Yangtze River Delta Urban Agglomeration in MCS and MRE Scenarios**

| Type | Scenario: MCS | | | Scenario: MRE | | |
| --- | --- | --- | --- | --- | --- | --- |
|  | 2025 | 2030 | 2035 | 2025 | 2030 | 2035 |
| Electricity generation for Shanghai (10^5^GWh) | | | | | | |
| Coal | 0.90 | 0.85 | 0.79 | 0.57 | 0.52 | 0.65 |
| Nature Gas | 0.03 | 0.03 | 0.04 | 0.00 | 0.00 | 0.00 |
| Nuclear | 0.00 | 0.00 | 0.00 | 0.00 | 0.00 | 0.00 |
| Hydro | 0.00 | 0.00 | 0.00 | 0.00 | 0.00 | 0.00 |
| Utility PV | 0.01 | 0.03 | 0.09 | 0.02 | 0.09 | 0.25 |
| Distributed PV | 0.03 | 0.08 | 0.15 | 0.05 | 0.11 | 0.34 |
| Onshore wind | 0.01 | 0.07 | 0.11 | 0.02 | 0.08 | 0.15 |
| Offshore wind | 0.14 | 0.24 | 0.53 | 0.14 | 0.28 | 0.66 |
| Biomass | 0.00 | 0.00 | 0.00 | 0.00 | 0.00 | 0.00 |
| Electricity generation for Jiangsu (10^5^GWh) | | | | | | |
| Coal | 4.35 | 4.05 | 3.88 | 3.97 | 3.57 | 3.12 |
| Nature Gas | 0.24 | 0.36 | 0.63 | 0.22 | 0.32 | 0.71 |
| Nuclear | 0.53 | 0.83 | 0.89 | 0.53 | 0.49 | 0.52 |
| Hydro | 0.03 | 0.06 | 0.10 | 0.03 | 0.07 | 0.10 |
| Utility PV | 0.26 | 0.62 | 1.27 | 0.39 | 1.02 | 1.58 |
| Distributed PV | 0.24 | 0.65 | 1.05 | 0.33 | 0.84 | 1.48 |
| Onshore wind | 0.25 | 0.49 | 0.96 | 0.26 | 0.82 | 1.22 |
| Offshore wind | 0.16 | 0.41 | 0.64 | 0.28 | 0.66 | 1.19 |
| Biomass | 0.29 | 0.27 | 0.29 | 0.29 | 0.27 | 0.29 |
| Electricity generation for Zhejiang (10^5^GWh) | | | | | | |
| Coal | 3.20 | 2.66 | 2.57 | 2.97 | 2.65 | 2.33 |
| Nature Gas | 0.15 | 0.28 | 0.59 | 0.07 | 0.10 | 0.77 |
| Nuclear | 0.73 | 0.83 | 0.94 | 0.73 | 0.71 | 0.88 |
| Hydro | 0.17 | 0.24 | 0.28 | 0.25 | 0.31 | 0.36 |
| Utility PV | 0.19 | 0.36 | 0.84 | 0.29 | 0.52 | 0.91 |
| Distributed PV | 0.13 | 0.43 | 0.76 | 0.23 | 0.49 | 0.75 |
| Onshore wind | 0.11 | 0.19 | 0.42 | 0.29 | 0.45 | 0.69 |
| Offshore wind | 0.04 | 0.09 | 0.17 | 0.04 | 0.14 | 0.32 |
| Biomass | 0.31 | 0.35 | 0.38 | 0.31 | 0.46 | 0.51 |
| Electricity generation for Anhui (10^5^GWh) | | | | | | |
| Coal | 2.88 | 2.76 | 2.64 | 2.59 | 2.47 | 2.24 |
| Nature Gas | 0.16 | 0.14 | 0.25 | 0.15 | 0.11 | 0.43 |
| Nuclear | 0.00 | 0.00 | 0.00 | 0.00 | 0.00 | 0.00 |
| Hydro | 0.05 | 0.14 | 0.19 | 0.05 | 0.25 | 0.19 |
| Utility PV | 0.33 | 0.59 | 1.05 | 0.49 | 0.91 | 1.58 |
| Distributed PV | 0.17 | 0.44 | 0.92 | 0.25 | 0.52 | 0.87 |
| Onshore wind | 0.46 | 0.86 | 1.28 | 0.56 | 0.85 | 1.12 |
| Offshore wind | 0.00 | 0.00 | 0.00 | 0.00 | 0.00 | 0.00 |
| Biomass | 0.18 | 0.20 | 0.22 | 0.19 | 0.20 | 0.22 |

**Supplementary Table 7. Relevant parameters of power generation technology**

| Type | Investment cost($/kW) | Maintenance cost（10^4^$/MW/a） | Carbon emission（t/MWh） | Lifetime(a) |
| --- | --- | --- | --- | --- |
| Hydro | 2080 | 3.70 | 0 | 50 |
| PV utility | 657 | 0.95 | 0 | 25 |
| PV distributed | 657 | 0.90 | 0 | 25 |
| Onshore wind | 1085 | 2.09 | 0 | 25 |
| Offshore wind | 2542 | 6.21 | 0 | 25 |
| Coal | 578 | 0.89 | 0.86 | 40 |
| Nuclear | 2285 | 7.95 | 0 | 50 |
| Gas | 341 | 1.40 | 0.31 | 40 |
| Biomass | 1504 | 6.4 | 0.35 | 40 |
| Energy storage | 457 | 2.28 | 0 | 15 |

**Supplementary Note 3:** **Construction and Operation scheme for Distributed Energy Stations**

Compared with the traditional decentralized energy supply system based on buildings, the centralized energy supply system can reduce the total installed capacity of the system, maintain the efficient operation of large cooling and heating equipment, while improving the quality of the buildings in the entire energy supply area. The gas-fired combined cooling, heating and power supply system has many advantages, such as high utilization rate of primary energy, low greenhouse gas emissions, safety and flexibility, as well as reducing the pressure on the power grid. With the potential for energy conservation and emission reduction, commercial circle and higher education zone have concentrated energy consumption, high and stable loads. In terms of energy consumption and load characteristics, it is suitable to adopt the construction of integrated energy stations to provide integrated energy services such as electricity, cooling, and heating for commercial circle and higher education zone, in order to achieve sustainable development of building energy consumption.

The energy station adopts a distributed electric cooling and heating triple supply system, which uses internal combustion engines to burn natural gas for power generation. At the same time, heat pumps and water storage technology are used for cooling and heating. Lithium bromide absorption refrigeration units are used to further recover and utilize the waste heat generated by the gas turbine after work.

Construction cost of distributed energy stations

Cost of purchasing equipment

Cost of purchasing generator unit

In order to reduce the cost of the generator unit, it is necessary to select the generator unit based on the energy demand of a single area. The alternative generator unit models and prices are shown in supplementary Table 8.

**Supplementary Table 8. Model and price of gas turbine**

| Manufacturer | Model | Rotating speed(r/m) | Price(10^6^$) | Power(MW) |
| --- | --- | --- | --- | --- |
| SOLAR | SATURN | 22120 | 0.8 | 1.08 |
| RUSTON | HURRICANE | 27245 | 1.1 | 1.58 |
| RUSTON | TB5000 | 7950 | 1.7 | 3.83 |
| SOLAR | TAURUS | 14950 | 1.9 | 4.37 |
| ALLISON | 571KA | 11500 | 2.8 | 5.59 |
| RUSTON | TORNADO | 11085 | 2.9 | 6.22 |
| SOLAR | MARS | 8568 | 4.3 | 8.84 |
| SOLAR | MARS | 9000 | 4.6 | 10.00 |
| MS | MF111A | 9660 | 5.8 | 12.84 |
| MS | MF111B | 9660 | 6.2 | 14.85 |
| GE | 5271RA | 5100 | 5.7 | 20.26 |
| RR | RB211 | 4800 | 11.1 | 25.25 |
| GE | 6541B | 5100 | 10.5 | 39.33 |
| WHL | 251 B12A | 5400 | 14.0 | 49.20 |
| KWU | V64.3 | 5400 | 18.5 | 60.65 |
| ABB | GT11N | 3600 | 20.5 | 81.60 |
| ABB | GT13D2 | 3000 | 22.5 | 100.50 |
| GE | 7191F | 3600 | 30.4 | 151.30 |
| KWU | V94.3 | 3000 | 41.0 | 200.36 |

Cost of control system

According to research, the cost of the control system for regional natural gas distributed energy supply projects in Shanghai is generally between 0.28 to 0.71 million dollar per unit, with an average value of 0.5 million dollar. The average installed capacity of the corresponding power generation device is 3.85MW, and the cost of the control system is calculated at 0.13 million dollar per MW.

Cost of gas and electric cooling and heating ancillary system equipment

According to research, the initial investment in gas regulator stations for regional natural gas distributed energy supply projects in Shanghai is between 0.3-0.9 million dollar, with a median of 0.4 million dollar, and the initial investment in electrical systems is between 0.7-2.1 million dollar, with a median of about 1 million dollar. The investment in gas supply system equipment for power generation projects is based on 0.1 million dollar per MW, and the investment in electrical system equipment is based on 0.25 million dollar per MW.

Cost of equipment installation

Cost of equipment installation accounts for generally 10% to 15% of the cost of purchasing generator unit, which is calculated as 12% in this paper.

Cost of construction

According to the current situation of distributed energy supply land in Shanghai, it is calculated at 0.08 million dollar per MW.

Other cost

Other cost include system debugging fees, engineering management fees, and design consulting fees, with system debugging fees and engineering management fees accounting for 4% of the total equipment price, design consulting fees accounting for 4.3% of the direct cost, and noise reduction and access fees considered at 0.14 million dollar per MW.

Interest during construction and operation period

The financing scheme is based on 20% of the principal and 80% of the bank loan. The construction period is two years, with 50% of loan ratio in the first year and 50% in the second year. The loan interest rate is 6.55%, the interest rate for the construction period is 0.12 million dollar per MW. The annual interest rate of the loan is calculated at 6.55%, the repayment period is ten years, of which the first three years is a grace period for repayment, only interest is paid but not the principal, from the fourth year the principal is repaid in equal amounts and interest is paid.

Operation and maintenance costs of distributed energy stations

The operating cost of energy stations mainly include fuel costs, wages, maintenance costs, insurance and land rentals, and their corresponding price standards are shown in supplementary Table 9.

**Supplementary Table 9. Standard for Operation and Maintenance**

**Costs of Distributed Energy Stations**

| Operating costs | | Standard |
| --- | --- | --- |
| Fuel costs | | 0.39 $/m^3^ |
| Wages | | 0.50 person/MW，25710 $/(person*a) |
| Maintenance costs | Repair costs | Repair cost of internal combustion engine is 0.01 $/kWh, and other equipment is charged at 2% of the original value of fixed assets |
|  | Materials | 2.14 $/MW |
|  | other costs | 2.57 $/MW |
| Insurance costs | | 0.25% of fixed asset investment |
| Land rentals | | 3571 $/(MW*a) |

Basic optimization model of distributed energy stations

The investment and operation strategy of the corresponding energy station is based on the cooling and heating energy demand of a single area to achieve the lowest total cost.

Objective function

The objective function includes the construction cost of the energy station and the revenue from operating the energy station. The construction cost includes the investment, operation, and maintenance costs of the energy station, and the revenue includes the revenue from the electricity, cooling, and heating produced by the energy station.

$$\text{R}_{DES}=R_{e}+R_{\text{h}}+R_{c}$$

where, C_DES_ is the total cost of investment in energy stations, R_DES_ is the total revenue from the operation of energy stations ,C*DES_I* is the investment costs of the energy station, C*DES_O&M* is the operation and maintenance costs of the energy station; R_e_ is the revenue from electricity sales, R_h_ is the revenue from heat sales; R_c_ is the revenue from cooling sales.

$$C_{DES\_I}=C_{DES\_e}+C_{DES\_i}+C_{DES\_c}+C_{DES\_o}$$

$$C_{\text{DES\_e}}=\sum_{type} \sum_{i=1}^{n_{i}} \sum_{j=1}^{n_{j}} \sum_{m=1}^{n_{m}} Cap_{m}^{i,j}\cdot p_{m,type}^{i,j}\cdot\delta_{m}\cdot N_{m},type\in\left\{unit,control,auxiliary\right\}$$

$$C_{DES\_i}+C_{DES\_c}+C_{DES\_o}=\sum_{type_{1}} \lambda_{type_{1}}\cdot\sum_{i=1}^{n_{i}} \sum_{j=1}^{n_{j}} \sum_{m=1}^{n_{m}} C\text{ap}_{m}^{i,j}+\sum_{type_{2}} \kappa_{type_{2}}\cdot C_{DES\_I}$$

$$type_{1}\in\left\{installation,other\right\},type_{4}\in\left\{construction,other\right\}$$

$$type_{3}\in\left\{salary,repairs,material,rent\right\},type_{4}\in\left\{insurance,other\right\}$$

where, C*DES_e* is the cost of purchasing generator unit, C*DES_i* is the cost of equipment installation, C*DES_c* is the cost of construction, C*DES_o* is other cost, C*DES_O&M* is the cost of operation and maintenance; $Cap_{m}^{i,j}$ is the installed capacity of the *m*-th type of generator unit in the *j*-th commercial circle or higher education zone in the *i*-th city, $p_{m}^{i,j}$ is the unit installed capacity price of the *m*-th type of generator unit in the *j*-th commercial circle or higher education zone in the *i*-th city, $P_{m,h}^{i,j}$、$P_{m,e}^{i,j}$、$P_{m,c}^{i,j}$ represents the power generation, heating, and cooling capacity of the *m*-th generation unit in the *j*-th commercial circle or higher education zone in the *i*-th city, respectively; *δ_m_* is 0 or 1; *N_m_*  is number of generation units of type *m; λ_type_* and *κ_type_* are coefficient of cost calculation; *p_NG_* is the price of nature gas; *η* is the energy use efficiency of the generating unit.

$$\lambda_{m,CHP}=P_{m,h}^{i,j}/P_{m,e}^{i,j}$$

$$P_{m,h}^{i,j}+P_{m,e}^{i,j}=Cap_{m}^{i,j}\cdot h_{m}^{i,j}\cdot N_{m}$$

$$P_{m,c}^{i,j}=\lambda_{hc}\cdot\eta_{hc}\cdot P_{m,h}^{i,j}$$

$$\text{R}_{DES}=\sum_{i=1}^{n_{i}} \sum_{j=1}^{n_{j}} \sum_{m=1}^{n_{m}} \left(P_{m,e}^{i,j}\cdot p_{e}+P_{m,h}^{i,j}\cdot p_{h}+P_{m,c}^{i,j}\cdot p_{c}\right)$$

where, *λ_m,CHP_* is the thermoelectric ratio of the m-th generator unit; $hour_{m}^{i,j}$ is the annual number of operating hours of the *m*-th type of generating unit in the *j*-th commercial circle or higher education zone in the *i*-th city; *λ_hc_* is the efficiency of waste heat refrigeration; *η_hc_* is the waste heat ratio used for refrigeration; *p_e_、p_h_、p*_c_ is the price of electricity, heat, and cooling.

Constraints

Constraints include thermoelectric ratio constraints, annual operating hours constraints, and energy constraints.

where, λ*_m_,*_CHP_*_min_* and λ*_m_*_,CHP_*_max_* are the upper and lower limits of the thermoelectric ratio for the *m*-th type of unit, respectively; and are the upper and lower limits of annual operating hours for the *m*-th type of unit, respectively; ,,,, and are the upper and lower limits of the electric cooling and heating output power of the *m*-th type of unit in the *j*-th commercial circle or higher education zone in the *i*-th city, respectively; and are the upper and lower limits of cooling demand for the *j*-th commercial circle or higher education zone; and are the upper and lower limits of heat demand for the *j*-th commercial circle or higher education zone; and are the upper and lower limits of the output of the *m*-th type of generator unit in the *j*-th commercial circle or higher education zone in the *i*-th city.

**Supplementary Note 4:** **Renovation Plan for Energy Infrastructure in Industrial Parks**

Emission reduction plan for greenhouse gases in industrial parks

S1: Transforming the extraction condensing or pure condensing unit of the steam turbine into a back pressure unit

According to the national energy strategy requirements, it is necessary to transform extraction or pure condensing units with installed capacity below 200MW into backpressure units^13^. The improvement of the condensing device is a technological upgrade of cogeneration. The working principle of backpressure units is to prioritize the generation of heat, while power generation is used as a supplement, which makes them have higher energy efficiency. On average, backpressure units are 12.4% and 19.2% higher than extraction condensing units and pure condensing units. Coal fired units with an installed capacity of less than 200MW and condensing technology of either extraction or pure condensing units are designated as units that can be retrofitted, the emission reduction of greenhouse gases in S1 scheme is calculated according to the following formula:

$$GHG\text{reduction}_{s1}=\sum_{i=1}^{n_{i}} \sum_{j=1}^{n_{j}} \sum_{k=1}^{n_{k}} GHGemission_{i,j}\cdot\left(1-\eta_{e,i,j,k}\div\frac{1+\lambda_{i,j,k}}{\frac{1}{\eta_{i,j,k}+\Delta\eta_{i,j,k}}+\frac{\lambda_{i,j,k}}{\eta_{i,j,k}}}\right)$$

where， GHGreduction_s1_ is the S1 scheme for greenhouse gas emissions reduction; GHGemission*_i,j,k_* is the greenhouse gas emissions of the *k*-th cogeneration unit in the *j*-th industrial park of the *i*-th city before renovation; *η_e,i,j_*, *λ_i,j,k_*, *η_i,j,k_*, Δ*η_i,j,k_* is the electrical efficiency, thermoelectric ratio, energy efficiency and improved energy efficiency of the *k*-th cogeneration unit in the *j*-th industrial park of the *i*-th city.

S2: Replacing coal-fired boilers with municipal solid waste incinerators

Most of municipal solid waste in China is disposed of in landfills, which leads to wasted land resources and serious environmental pollution^14^. The Chinese government has promoted municipal solid waste incineration in the 13th Five Year Plan, which is also widely adopted in industrial parks in China. The total installed capacity of municipal solid waste incineration in industrial parks in China is 2.4GW, accounting for 55% of the total capacity. Previous research has shown that the installed capacity of solid waste incineration devices is 3-30MW, and use them for power generation and heating can improve environmental performance^16^. We use municipal solid waste incinerators to replace coal-fired boilers in industrial parks to achieve energy conservation and emission reduction.

The installed capacity of municipal solid waste incinerators in China in 2014 was 4.3GW, and in 2020 it was 7.5GW^15^. Based on this growth rate, the installed capacity of municipal solid waste incinerators in China will be 31.3GW by 2035. Assuming that the proportion of municipal solid waste incinerators installed capacity in industrial parks remains unchanged at around 50%, the installed capacity of municipal solid waste incinerators in 2035 is estimated based on the proportion of existing municipal solid waste incinerators installed capacity in the Yangtze River Delta, in order to determine the quota for cities within the Yangtze River Delta urban cluster by 2035, The newly added municipal solid waste incinerators installed capacity for industrial parks in Shanghai is 136MW, 1184MW in Jiangsu Province, 670MW in Zhejiang Province, and 502MW in Anhui Province.

Coal fired units with installed capacity of 3-30MW are defined as units that need to be retrofitted. Compared to small units, larger units have higher thermal efficiency and better energy-saving and emission reduction effects. Therefore, priority is given to retrofitting small capacity coal fired units to municipal solid waste incinerators. Usually, waste incinerators require the addition of coal for mixed combustion, with a coal proportion ranging from 10% to 20%.

The emission reduction of greenhouse gases under the S2 scheme is calculated using the following formula:

$$GHG\text{reduction}_{s2}=\sum_{i=1}^{n_{i}} \sum_{j=1}^{n_{j}} \sum_{k=1}^{n_{k}} GHGemission_{i,j,k}\cdot\left(1-\frac{\eta_{i,j,k}}{\eta_{MSW}}\cdot\frac{\left(1-\alpha\right)\cdot GHG\text{fac}_{MSW}+\alpha\cdot GHG\text{fac}_{coal}}{GHG\text{fac}_{coal}}\right)$$

where, GHGreduction_s2_ is the S2 scheme for greenhouse gas emissions reduction; GHGemission_i,j,k_ and *η_i,j,k_* is greenhouse gas emissions and energy efficiency of the *k*-th coal-fired unit in the *j*-th industrial park of the *i*-th city before renovation, respectively; *η_MSW_* is the energy efficiency of municipal solid waste incinerators; $\alpha$ is the proportion of coal added for mixed combustion; GHGfac_MSW_ and GHGfac_coal_ are greenhouse gas emission coefficient of coal-fired units and municipal solid waste incinerators, respectively.

S3: Replacing coal-fired boilers with gas turbines.

The energy infrastructure of industrial parks heavily relies on coal, with 80.5% of the units in the Yangtze River Delta urban agglomeration being coal-fired. Natural gas is China's energy strategy choice and should be fully utilized under limited quotas. Compared to coal-fired power, gas power has a higher electricity price. The emission reduction of greenhouse gases under the S3 scheme is calculated using the following formula:

$$GHG\text{reduction}_{s3}=\sum_{i=1}^{n_{i}} \sum_{j=1}^{n_{j}} \sum_{k=1}^{n_{k}} GHGemission_{i,j,k}\cdot\left(1+\frac{1-\left(1+\lambda_{i,j,k}\right)\cdot\eta_{NG}\cdot GHG\text{fac}_{NG}}{\lambda_{i,j,k}\cdot GHG\text{fac}_{\text{coal}}}\right)$$

where, GHGreduction_s3_ is the S3 scheme for greenhouse gas emissions reduction; *η*_NG_ is the energy efficiency of gas turbine units; GHGfac_NG_ is the greenhouse gas emission coefficient of coal-fired units.

Retrofit cost analysis of GHG reduction options for industrial estates

The renovation costs for scheme S1 and scheme S2 are calculated at 10428 dollar per MW and 175571 dollar per MW, respectively. Scheme S3 requires the reconstruct of the infrastructure of the industrial park, and the optimization of its unit commitment, operation and maintenance strategy can further reduce the cost, which is similar to the construction of distributed energy stations in the commercial circle and the high education zone, except that the scheme for the industrial park uses cogeneration technology, while the scheme for the commercial circle and the high education zone uses cooling, heating and electricity trigeneration technology, and the optimization model for the construction of gas-fired units in the industrial park is as follows:

According to the heat load of individual industrial parks, the investment and operation strategy of the corresponding gas-fired unit is developed to achieve the lowest total cost. The objective function includes the construction costs of the gas-fired units and the benefits of their operation, where the construction costs include the investment, operation and maintenance costs of the gas-fired units and the benefits include the benefits of the electricity and heat produced by the gas-fired units.

$$\text{Revenue}=R_{e}+R_{\text{h}}$$

where, C*I_I* is the investment cost of energy station; C*I_O&M* is the operation and maintenance costs of the energy station; R_e_ is the revenue from electricity sales, R_h_ is the revenue from heat sales.

$$C_{I\_i}=C_{I\_e}+C_{I\_i}+C_{I\_c}+C_{I\_o}$$

$$C_{\text{I\_e}}=\sum_{type} \sum_{i=1}^{n_{i}} \sum_{j=1}^{n_{j}} \sum_{m=1}^{n_{m}} Cap_{m}^{i,j}\cdot p_{m,type}^{i,j}\cdot\delta_{m}\cdot N_{m},type\in\left\{unit,control,auxiliary\right\}$$

$$C_{I\_i}+C_{I\_c}+C_{I\_o}=\sum_{type_{1}} \lambda_{type_{1}}\cdot\sum_{i=1}^{n_{i}} \sum_{j=1}^{n_{j}} \sum_{m=1}^{n_{m}} Cap_{m}^{i,j}+\sum_{type_{2}} \kappa_{type_{2}}\cdot C_{I\_I}$$

$$type_{1}\in\left\{installation,other\right\},type_{4}\in\left\{construction,other\right\}$$

$$type_{3}\in\left\{salary,repairs,material,rent\right\},type_{4}\in\left\{insurance,other\right\}$$

where, C*I_e* is the cost of purchasing generator unit, C*I_i* is the cost of equipment installation, C*I_c* is the cost of construction, C*I_o* is other cost, C*I_O&M* is the cost of operation and maintenance; $Cap_{m}^{i,j}$ is the installed capacity of the *m*-th type of generator unit in the *j*-th industrial park in the *i*-th city, $p_{m}^{i,j}$is the unit installed capacity price of the *m*-th type of generator unit in the *j*-th industrial park in the *i*-th city, $P_{m,h}^{i,j}$、$P_{m,e}^{i,j}$represents the power generation and heating capacity of the *m*-th generation unit in the *j*-th industrial parks in the *i*-th city, respectively; *δ_m_* is 0 or 1; *N_m_*  is number of generation units of type *m; λ_type_* and *κ_type_* are coefficient of cost calculation; *p_NG_* is the price of nature gas; *η* is the energy use efficiency of the generating unit.

$$\lambda_{m,CHP}=P_{m,h}^{i,j}/P_{m,e}^{i,j}$$

$$P_{m,h}^{i,j}+P_{m,e}^{i,j}=Cap_{m}^{i,j}\cdot h_{m}^{i,j}\cdot N_{m}$$

$$\text{Revenue}=\sum_{i=1}^{n_{i}} \sum_{j=1}^{n_{j}} \sum_{m=1}^{n_{m}} \left(P_{m,e}^{i,j}\cdot p_{e}+P_{m,h}^{i,j}\cdot p_{h}\right)$$

where, *λ_m,CHP_* is the thermoelectric ratio of the *m*-th generator unit; $h_{m}^{i,j}$ is the annual number of operating hours of the *m*-th type of generating unit in the *j*-th industry park in the *i*-th city; *p_e_* and *p_h_* is the price of electricity and heat.

The constraints include thermoelectric ratio constraints, annual operating hours constraints, and energy constraints.

where, λ*_m_,*_CHP_*_min_* and λ*_m_*_,CHP_*_max_* are the upper and lower limits of the thermoelectric ratio for the *m*-th type of unit, respectively; and are the upper and lower limits of annual operating hours for the *m*-th type of unit, respectively; ,,, and are the upper and lower limits of the electric and heating output power of the *m*-th type of unit in the *j*-th industry park in the *i*-th city, respectively; and are the upper and lower limits of thermal demand for industry park, respectively; and are the upper and lower limits of the output of the *m*-th type of generator unit in the *j*-th industry park in the *i*-th city.

**Supplementary Table 10. Thermal power ratio of cogeneration units classified by capacity**

| Install capacity/MW | Min | Max |
| --- | --- | --- |
| ≥600 | 0.0003 | 0.744 |
| [300,600] | 0.0017 | 1.669 |
| [100,300] | 0.0532 | 3.480 |
| ＜100MW | 0.4838 | 14.964 |

**Supplementary Table 11. Greenhouse gas emission coefficients for various energy sources**

| Fuel | Coal | NG | MSW | Biomass | Diesel |
| --- | --- | --- | --- | --- | --- |
| tCO_2_/GJ | 0.0952 | 0.0556 | 0.0330 | 0.0019 | 0.0728 |

**Supplementary Table 12. Electrical efficiency and annual operating hours of energy infrastructure classified by fuel, capacity, and technology**

| Fuel | Capacity and technology | Effective electric efficiency | | Annual working hours | |
| --- | --- | --- | --- | --- | --- |
|  |  | min | max | min | max |
| Coal | PC,＜100 | 0.130 | 0.433 | 117 | 8859 |
|  | PC,[100,300) | 0.296 | 0.431 | 535 | 7226 |
|  | PC,[300,600) | 0.334 | 0.442 | 2045 | 8352 |
|  | PC，≥600 | 0.346 | 0.460 | 1237 | 7323 |
|  | EC,＜100 | 0.121 | 0.793 | 140 | 9875 |
|  | EC,[100,300) | 0.271 | 0.585 | 1223 | 9111 |
|  | EC,[300,600) | 0.253 | 0.532 | - | - |
|  | EC,＞600 | 0.375 | 0.434 | 3556 | 7128 |
|  | BP | 0.228 | 0.884 | 355 | 8760 |
| NG | NGCC | 0.302 | 0.690 | 232 | 7633 |
|  | PC/EC | 0.352 | 0.654 | - | - |
| MSW | PC | 0.137 | 0.378 | 858 | 8766 |
|  | EC | 0.156 | 0.679 | 610 | 9223 |

References

[1] Brooker, A., Gonder, J., Lopp, S., and Ward, J. ADOPT: A Historically Validated Light Duty Vehicle Consumer Choice Model. SAE Technical Paper 2015-01-0974, 2015, doi:10.4271/2015-01-0974.

[2] BloombergNEF. Battery Pack Prices Cited Below $100/kWh for the First Time in 2020, While

Market Average Sits at $137/kWh (BloombergNEF, 2020, Retrieved February 5, 2021 from [https://about.bnef.com/blog/battery-pack-prices-cited-below-100-kwh-for-the-first-time-in-2020-while-market-average-sits-at-137-kwh/.](https://about.bnef.com/blog/battery-pack-prices-cited-below-100-kwh-for-the-first-time-in-2020-while-market-average-sits-at-137-kwh/)

[3] Goldie-Scot, L. A Behind the Scenes Take on Lithium-ion Battery Prices (BloombergNEF, 2019, Retrieved June 23, 2020 from <https://about.bnef.com/blog/behind-scenes-take-lithium-ion-battery-prices.>

[4] DOE, forthcoming. Estimates provided from communications with DOE

[5]National Energy Administration. Guidelines on accelerating the construction of charging facilities for electric vehicles(2015). <http://www.gov.cn/zhengce/content/2015-10/09/content_10214.htm>

[6]National Energy Administration. Guidelines on accelerating the construction of charging facilities for electric vehicles(2015). <http://www.nea.gov.cn/2015-11/18/c_134828653.htm>

[7]Notice on the financial subsidy policy for the promotion and application of new energy vehicles in 2022. Ministry of finance of the people's Republic of China.

[8]Lu, Tianwei, Enjian Yao, Fanglei Jin, and Yang Yang. Analysis of Incentive Policies for Electric Vehicle Adoptions after the Abolishment of Purchase Subsidy Policy. Energy (Oxford) 239 (2022): 122136.

[9] Guangzhou Municipal People's Government. Guidelines for Encouraging and Supporting the Promotion and Application of New Energy Vehicles in Personal Fields in Guangzhou City.(2023) <https://www.gz.gov.cn/zwfw/zxfw/jtfw/content/post_8900861.html>

[10]Jung, Yujun, Joonbyum Kim, and Hoseong Lee. Multi-criteria Evaluation of Medium-sized Residential Building with Micro-CHP System in South Korea. Energy and Buildings 193 (2019): 201-15.

[11]Mago, Pedro J., and Amanda D. Smith. Evaluation of the Potential Emissions Reductions from the Use of CHP Systems in Different Commercial Buildings. Building and Environment 53 (2012): 74-82.

[12]<https://www.sohu.com/a/101737313_229282>

[13]U.S. Environmental Protection Agency. Catalog of CHP technologies. (2015). <http://www.epa.gov/sites/production/files/2015-07/documents/catalog_of_chp_technologies.pdf.>

[14]National Development and Reform Committee of China. Action plan for energy saving, emission reduction, upgrading and alteration of coal-fired electricity generation (2014-2020). (2014). <http://www.zhb.gov.cn/gkml/hbb/gwy/201409/W020140925407622627853.pdf>.

[15]Zheng, L. et al. Preferential policies promote municipal solid waste (MSW) to energy in China: Current status and prospects. Renewable & Sustainable Energy Reviews 36, 135-148 (2014).

[16]National Energy Administration of China. The 13th five-year plan of bioenergy development. (2017). <http://ghs.ndrc.gov.cn/ghwb/gjjgh/201708/t20170809_857319.html>.

[17]Guo, Y., Glad, T., Zhong, Z., He, R., Tian, J. & Chen, L. Environmental life-cycle assessment of municipal solid waste incineration stocks in Chinese industrial parks. Resources, Conservation & Recycling 139, 387-395
